# Stromal Cells Positively and Negatively Modulate the Growth of Cancer Cells: Stimulation via the PGE2-TNFα-IL-6 Pathway and Inhibition via Secreted GAPDH-E-Cadherin Interaction

**DOI:** 10.1371/journal.pone.0119415

**Published:** 2015-03-18

**Authors:** Manabu Kawada, Hiroyuki Inoue, Shun-ichi Ohba, Junjiro Yoshida, Tohru Masuda, Manabu Yamasaki, Ihomi Usami, Shuichi Sakamoto, Hikaru Abe, Takumi Watanabe, Takao Yamori, Masakatsu Shibasaki, Akio Nomoto

**Affiliations:** 1 Institute of Microbial Chemistry (BIKAKEN), Numazu, Microbial Chemistry Research Foundation, Numazu-shi, Shizuoka, Japan; 2 Institute of Microbial Chemistry (BIKAKEN), Tokyo, Microbial Chemistry Research Foundation, Shinagawa-ku, Tokyo, Japan; 3 The Cancer Chemotherapy Center, Japanese Foundation for Cancer Research, Koto-ku, Tokyo, Japan; Mie University Graduate School of Medicine, JAPAN

## Abstract

Fibroblast-like stromal cells modulate cancer cells through secreted factors and adhesion, but those factors are not fully understood. Here, we have identified critical stromal factors that modulate cancer growth positively and negatively. Using a cell co-culture system, we found that gastric stromal cells secreted IL-6 as a growth and survival factor for gastric cancer cells. Moreover, gastric cancer cells secreted PGE2 and TNFα that stimulated IL-6 secretion by the stromal cells. Furthermore, we found that stromal cells secreted glyceraldehyde 3-phosphate dehydrogenase (GAPDH). Extracellular GAPDH, or its N-terminal domain, inhibited gastric cancer cell growth, a finding confirmed in other cell systems. GAPDH bound to E-cadherin and downregulated the mTOR-p70S6 kinase pathway. These results demonstrate that stromal cells could regulate cancer cell growth through the balance of these secreted factors. We propose that negative regulation of cancer growth using GAPDH could be a new anti-cancer strategy.

## Introduction

Tumor tissues are composed of cancer cells and surrounding stroma. The stroma contains various types of cells embedded in a matrix such as macrophages, endothelial cells, immune cells, and fibroblast-like stromal cells [1, 2]. The stromal cells exhibit phenotypic plasticity changing between fibroblastic and myofibroblastic characteristics [3, 4]. Myofibroblasts expressing vimentin and smooth muscle α-actin (SM-α-actin) [1] have a high ability to secrete growth factors, ECM proteins, and proteinases [1, 5] and are termed reactive stroma or cancer-associated fibroblasts (CAFs) [5, 6]. Since the myofibroblast content of tumor tissues correlates well with poor prognosis of some cancers [7], stromal cells, especially myofibroblasts, are significantly involved in the development of cancer. Recent reports have clarified the role of stromal cells in the maintenance of cancer stem cells [8], metastatic niches [9, 10], and chemoresistance [11, 12]. Due to such growing evidence, stromal cells are becoming an attractive target for anti-cancer strategies.

Stromal cells regulate the growth of cancer cells positively and negatively through secreted factors and adhesion [1, 5, 6, 13–17]. Various growth factors such as hepatocyte growth factor (HGF) [18], insulin-like growth factor-I (IGF-I) [19], and fibroblast growth factor-7 (FGF-7) [20] are secreted from stromal cells. On the other hand, cancer cells also secrete various factors to modify or “educate” stromal cells to improve their microenvironment. Transforming growth factor-β1 (TGF-β1) is one of such factors and stimulates fibroblasts to differentiate into myofibroblasts [1, 3]. Theses factors and interactions between cancer cells and stromal cells differ in each cancer and thus are not fully understood [21].

We have been studying tumor-stromal cell interactions using co-culture systems of both cells [22]. While prostate stromal cells increase the growth of prostate cancer cells when co-injected into nude mice [19], our *in vitro* co-culture method mimics the *in vivo* results [22]. Using this model, we have found that IGF-I is secreted from prostate stromal cells and plays a critical role in prostate cancer development [19]. Furthermore, we used the *in vitro* co-culture system as a screening assay to identify compounds that exert anti-cancer activity through the modulation of tumor-stromal cell interactions. As a result, we have discovered many compounds from natural sources such as cultured broths of bacteria and fungi [23–26]. Among them, phthoxazolin A and leucinostatin A are found to inhibit the secretion of IGF-I from prostate stromal cells and suppress the growth of prostate cancer cells in the presence of stromal cells [23, 24]. On the other hand, NBRI16716A inhibits the growth of prostate cancer cells in a xenograft model [26], but it does not affect the secretion of IGF-I from prostate stromal cells. Our preliminary experiments suggest that NBRI16716A might stimulate stromal cells to secrete unidentified tumor suppressive factors. Thus, these results strongly indicate that we can control the cancer development by the modulation of tumor-stromal cell interactions.

In this study, we examined the interactions using gastric cancer as a model. We have identified critical factors that modulate the growth of cancer cells positively and negatively. These findings suggest new anti-cancer strategies.

## Materials and Methods

### Cell lines and reagents

Human prostate cancer DU-145 cells, human colon cancer DLD-1 cells, human pancreatic cancer cell lines MiaPaca2, BxPC-3, Capan-1 and Panc-1 were obtained from the American Type Culture Collection (ATCC). Human prostate cancer PC-3 cells and human embryonic kidney 293 cells were obtained from DS Pharma. The LNCaP-CR cell line [27] was established in our laboratory from human prostate cancer LNCaP cells (DS Pharma). Other cancer cell lines were described elsewhere [28, 29]. All cancer cell lines were maintained in Dulbecco’s Modified Eagle’s Medium (DMEM) (Nissui) supplemented with 10% fetal bovine serum (FBS; Sigma), 100 units/mL penicillin G (Invitrogen), and 100 μg/mL streptomycin (Invitrogen) at 37°C with 5% CO_2_. Hs738 human gastric stromal cells (CRL-7869), CCD-18Co human colon fibroblasts (CRL-1459), and Hs371 mammary gland fibroblasts (CRL-7256) were obtained from the ATCC. NHLF normal human lung fibroblasts and PrSC human prostate stromal cells were obtained from BioWhittaker. PS human pancreatic stromal cells were obtained from DS pharma. All stromal cells were maintained in DMEM supplemented with 10% FBS, 100 units/mL penicillin G, 100 μg/mL streptomycin, ITH (5 μg/mL insulin, 5 μg/mL transferrin, and 1.4 μM hydrocortisone), and 5 ng/mL basic FGF (PeproTech) at 37°C with 5% CO_2_ as described [22].

Anti-pan-cytokeratin (sc-8018), anti-STAT3 (sc-8019), anti-GAPDH (sc-47724), anti-PAI-1 (sc-8979), anti-p70S6 kinase (sc-230), anti-14–3–3 epsilon (sc-1020), and anti-phospho-MAPK (sc-7383) antibodies were purchased from Santa Cruz Biotechnology. Anti-vimentin (V2258), anti- SM-α-actin (A2547), anti-α-tubulin (T9026), anti-phospho-(tyr705)-STAT3 (SAB4300033), anti-RPL-18A (HPA055259), and anti-FLAG M2 (F3165) antibodies, rabbit muscle GAPDH (G2267) and human erythrocyte GAPDH (G6019) were purchased from Sigma. Anti-phospho-Ser/Thr kinase substrate (9614 and 9624), anti-ribosomal protein S6 (RPS6) (2217), anti-phospho-(Ser235/236)-RPS6 (2211), anti-phospho-(Ser240/244)-RPS6 (2215), anti-phospho-(Ser473)-Akt (9271), anti-phospho-(Thr389)-p70 S6 kinase (9234), anti-phospho-(Tyr416)-Src family (2102), anti-phospho-(Thr172)-AMPKα (2535), anti-Myc (2278), anti-caveolin-1 (3267), and anti-β-catenin (9562) antibodies were purchased from Cell Signaling Technology. Anti-phospho-14–3–3 antibody was purchased from Abgent. Anti-phospho-(Tyr705)-STAT3 (612356) antibody was purchased from BD Biosciences. Anti-RPL-18A antibody was purchased from Abcam. Anti-human IL-6 neutralizing antibody (MAB206), recombinant human IL-6 (206-IL), and recombinant human CXCL1 (275-CR/CF) were purchased from R&D Systems. Anti-human CXCL1 neutralizing antibody (LS-C104351) was purchased from Lifespan Biosciences. Anti-α-enolase (MO1) antibody and human recombinant GAPDH (P4547) were purchased from Abnova. Anti-mouse IgG1 Alexa Fluor 546, anti-mouse IgG2a Alexa Fluor 546, and anti-mouse IgG1 Alexa Fluor 350 antibodies were purchased from Invitrogen. Anti-E-cadherin (SHE78–7) antibody was purchased from Enzo Life Science.

Small interfering RNAs (siRNA) targeting RPS6 were generated as RPS6 Si#1, 5’-CUGCGAGCUUCUACUUCUAAG-3’ and RPS6 Si#2, 5’-GACUGACUGAUACUACAGUGC-3’ and purchased from Sigma with control siRNA. ON-TARGETplus SMARTpool siRNAs against human E-cadherin and RPL-18A and control siRNA, ON-TARGETplus Non-targeting pool, were purchased from Thermo Scientific. The siRNAs were transfected using lipofectamine 2000 or lipofectamine RNAiMAX reagents (Invitrogen) according to the manufacturer’s protocol.

SCADS inhibitor kits I, II, and III (about 300 small molecule inhibitors) were kindly provided by the Screening Committee of Anticancer Drugs supported by Grant-in-Aid for Scientific Research on Innovative Areas, Scientific Support Programs for Cancer Research, from The Ministry of Education, Culture, Sports, Science and Technology, Japan. PD98059, U0126, MEK inhibitor I, MEK inhibitor II, MEK1/2 inhibitor II, and MEK1/2 inhibitor I were purchased from Merck. PGE2 was purchased from Cayman. Tissue arrays of human gastric cancer (A209II) were purchased from ISU ABIXS.

### GFP transfection

Cells (2 x 10^5^) were cultured in 6-well plates in 10% FBS-DMEM for 2 days. The medium was replaced with 800 μL of OPTI-MEM (Invitrogen), and then 200 μL of OPTI-MEM containing 1 μg of pEGFP-C1 vector (BD Biosciences), 4 μL of Lipofectamine Reagent (Invitrogen) and 6 μL of PLUS Reagent (Invitrogen) were added to the cells. After a 3 h incubation, 1 mL of 20% FBS-OPTI-MEM was added to each well and the cells were cultured for 24 h. Stably transfected cells were selected with 400 μg/mL of G418 (Promega).

### Preparation of cell lysates and western blotting

The cell lysates were prepared and directly applied to Western blots or immunoprecipitated with antibodies as described [30].

### Immunofluorescence

Cells were cultured on glass cover slips in a 6-well plate at 1 x 10^5^ cells per well in DMEM supplemented with 1% dialyzed-FBS (D-FBS) prepared by dialysis against phosphate-buffered saline (PBS) and ITH. The cells were washed with PBS and fixed with cold acetone for 2 min. Hs738 cells were stained with anti-vimentin and SM-α-actin antibodies as described [4]. Gastric cancer cells were stained with anti-phospho-(Ser235/236)-RPS6 antibody and then with secondary anti-rabbit IgG Alexa Fluor 546 (Invitrogen). For GAPDH immunostaining, cells were fixed with 4% formaldehyde in PBS for 15 min for nonpermeabilized conditions or 4% formaldehyde in PBS for 15 min and then methanol for 5 min for permeabilized conditions. The fixed cells were stained with anti-FLAG, anti-GAPDH, or anti-E-cadherin and then with anti-mouse IgG_1_ Alexa Fluor 546, anti-mouse IgG_2a_ 546, or anti-mouse IgG_1_ 488. The stained cells were analyzed with a Leica DM IRB fluorescence microscope or an Olympus FV300 laser scanning confocal microscope with FluoView software (Olympus).

### Animal experiments

All animal experiments were approved by the Institutional Committee for Animal Experiments in Institute of Microbial Chemistry and performed in accordance with relevant guidelines and regulations to minimize animal suffering. Female nude mice, 6 weeks old, were purchased from Charles River Breeding Laboratories and maintained in a specific pathogen-free barrier facility according to our institutional guidelines. The mice at 7 to 8 weeks of age were used for experiments. Gastric cancer cells (8 x 10^6^) were trypsinized and resuspended with or without Hs738 cells (8 x 10^6^) in 0.3 mL of 10% FBS-DMEM and then combined with 0.5 mL of growth factor-reduced Matrigel (BD Biosciences). One hundred microliters of the cell suspension (1 x 10^6^ cells) were injected subcutaneously in the left flank of the mice. Six mice were used for each experimental set. Tumor volume was estimated using the following formula: tumor volume (mm^3^) = (length x width^2^)/2. After the indicated days, tumors were surgically dissected. For orthotopic implantation, female mice were anesthetized by intraperitoneal injection of pentobarbital sodium at 66.7 mg/kg. After making a small median abdominal incision, gastric cancer cells (1 x 10^6^) in 20 μL of the Matrigel-containing medium were inoculated into the middle wall of the greater curvature of the glandular portion of the stomach using a 30-gauge needle (Nipro) [31]. The stomach was returned into the peritoneal cavity, and the abdominal wall was sutured and the skin was closed with an AUTOCLIP applier (Becton-Dickinson). Mice were euthanized on the indicated days after the tumor inoculation.

### Cell growth and co-culture experiments

Cells were inoculated into a 96-well plates at 5 x 10^3^ cells per well in 0.1 mL of DMEM supplemented with 1% D-FBS and ITH. After culture for the indicated days, cell growth was determined using 3-(4,5-dimethyllythiazol-2-yl)-2,5-diphenyltetrazolium bromide (MTT; Sigma) as described [32] or measuring GFP fluorescence intensity (excitation at 485 nm and emission at 538 nm) by the lysis of cells in 10 mM Tris-HCl pH7.4, 150 mM NaCl, 0.9 mM CaCl_2_, and 1% Triton X-100. For co-culture experiments, Hs738 cells were first inoculated into 96-well plates at 5 x 10^3^ cells per well in 0.1 mL of DMEM supplemented with 1% D-FBS and ITH. Test samples were added to the wells and the cells were cultured for 2 days. Then, 10 μL of a gastric cancer cell suspension (5 x 10^3^) in serum-free DMEM were added to the monolayer of Hs738 cells and the cells were further cultured for 3 days. For monoculture of gastric cancer cells, assay medium alone with a test sample was first incubated for 2 days, and then gastric cancer cells were added as described above and further cultured for 3 days. The growth of gastric cancer cells was determined measuring GFP fluorescence intensity. For suspension culture, cells were inoculated into a 96-well suspension culture plate (MS-8096R; Sumilon) for comparison with a standard culture plate (MS-8096F; Sumilon). For Transwell experiments, 0.6 mL of Hs738 cells (5 x 10^4^ cells/mL) in DMEM supplemented with 1% D-FBS and ITH were first added to outer wells of Transwell plates with 0.4 μm pore size membranes (3470; Corning) and 0.1 mL of the assay medium was added to the inner wells. After 2 days of culture, 10 μL of a gastric cancer cell suspension (5 x 10^3^) in serum-free DMEM was added to the inner wells and the plates were further cultured for 3 days.

### Preparation of Hs738 conditioned medium (CM)

Hs738 cells were cultured at 5 x 10^4^ cells/mL in DMEM supplemented with the indicated concentrations of D-FBS and ITH for the indicated days. The cultured supernatants were collected by centrifugation. To concentrate the medium, Amicon Ultra-15-3K centrifugal filter devices (Millipore) or 2-D clean-up kit (Amersham Biosciences) were used. Gastric cancer cells (3 x 10^5^) were inoculated into 1 mL of the CM of Hs738 cells or assay medium alone in 35 mm dishes and cultured for 1 day. The cells were washed with PBS and the cell lysates were prepared for Western blotting.

### Proteome analysis of CM

For the identification of phosphorylated proteins from gastric cancer cells, MKN-7 cell lysates were applied onto a HiTrap Q FF column (GE Healthcare) preloaded with 50 mM Tris-HCl pH7.3. Proteins were eluted by stepwise increases of NaCl concentrations. After elution, protease inhibitors and pyrophosphate were added to the eluted fractions. The eluted fractions were separated by SDS-PAGE, and the gels were stained with Coomassie brilliant blue (CBB) and bands identical to the bands of Western blots obtained with anti-phospho-Ser/Thr antibody were excised. The excised bands were digested with trypsin, and the digested peptides were then subjected to LC-MS/MS analysis (LTQ-Orbitrap; Thermo Scientific). For the identification of growth-inhibitory proteins in Hs738 CM, the CM (50 mL) was concentrated using Amicon Ultra-15-3K centrifugal filter devices and applied to a Superdex 200 10/300 GL column (GE Healthcare) for gel filtration. Proteins were eluted by PBS and the effect of the fractioned proteins on growth of MKN-7 cells was determined. The fractions with growth inhibitory activity were pooled and further separated by 2D gel electrophoresis. Spots of interest were excised, digested with trypsin, and then subjected to LC-MS/MS or MALDI-TOF-MS analysis (APRO Life Science Institute).

### Cytokine analysis

Cells were cultured at 5 x 10^4^ cells/mL in DMEM supplemented with 1% D-FBS and ITH for 2 days. The CMs were collected by centrifugation and the amounts of IL-6, IL-6sR, and CXCL1 were determined using a human IL-6 ELISA kit (Thermo Scientific), a human IL-6sR Quantikine (R&D Systems) and a human CXCL1 Quantikine (R&D Systems). The culture supernatants were also applied onto human cytokine antibody array (C series 2000; RayBio). The amounts of PGE2, 6-keto PG, thromboxian B_2_, and leukotriene B4 were determined using quantitative kits from Cayman.

### Immunohistochemistry

Tissue arrays were deparaffinized in xylene and rehydrated in descending alcohols into water. Then, the tissue arrays were dipped in 3% H_2_O_2_ for 13 min and boiled in 10 mM citric acid (pH 6.0) for 15 min. Immunohistochemical staining was conducted with anti-phospho-(Tyr705)-STAT3 (SAB43000033) using ImmPRESS anti-rabbit Ig peroxidase (Vector Laboratories) and ImmPACT DAB substrate (Vector Laboratories). The estimated visual intensity of phospho-STAT3 immunostaining was graded on an arbitrary 3 point scale: negative, weakly positive, and positive.

### GAPDH activity

GAPDH activity was measured as described [33]. All reagents were purchased from Sigma.

### Construction of recombinant GAPDH

Human recombinant wild-type GAPDH and mutant GAPDH were constructed as described [34], with modifications as follow. Total RNA was isolated from Hs738 cells using the RNeasy Minikit (Qiagen). The cDNA was synthesized using AMV reverse transcriptase (Promega) and the full-length human GAPDH cDNA fragment containing *Nhe*I and *Xho*I sites was generated by PCR using PfxDNA polymerase (Invitrogen) and sense primer 1 (5’-TATGCTAGCGACTACAAGGACGACGACGACAAGATGGGGAAGGTGAAGGTCGGAG-3’) and antisense primer 2 (5’-TATCTCGAGTTACTCCTTGGAGGCCATGTGGG-3’). The *Nhe*I/*Xho*I fragment of GAPDH was inserted into pET-17b vector (Novagen). For mutant GAPDH (C152S), fragment 1 was generated by PCR using sense primer 1 and antisense primer 3 (5’-AGGGGTGCTAAGCAGTTGGTGGTGGAGGAGGCATTGCTGATGATCTTGA-3’) and fragment 2 was generated by PCR using sense primer 4 (5’-TCAAGATCATCAGCAATGCCTCCTCCACCACCAACTGCTTAGCACCCCT-3’) and antisense primer 2. The full-length mutant GAPDH fragment was generated by PCR using sense primer 1, antisense primer 2, and fragments 1 and 2 as templates. The *Nhe*I/*Xho*I fragment of mutant GAPDH was also inserted into pET-17b vector. Deletion mutants of GAPDH were constructed by inverse PCR using a KOD-Plus-mutagenesis kit (Toyobo) and primer sets for del1, 5’-gcagttggtggtgcaggaggcatt-3’ and 5’-gacaacgaatttggctacagcaac-3’, for del2, 5’-ATCAAGTGGGGCGATGCTGGCGCTGAGTA-3’ and 5’-CTTCCCCATCTTGTCGTCGTCGTCCTT-3’, and for del3, 5’-CTCCACGACGTACTCAGCGCCAG-3’ and 5’-accaccaactgcttagcacccctg-3’. The vectors were transfected into BL21(DE3) *E*. *coli* (Promega) or ClearColiBL21 (Lucigen) and N-terminal FLAG-tagged human wild-type and mutant GAPDH were purified using anti-FLAG M2 agarose (Sigma). The partially purified GAPDH was further purified by gel filtration (GE Healthcare) and endotoxin removal resin (Thermo Scientific).

### Binding of GAPDH to cell membranes

Cell membranes were prepared using ProteoExtract kit (Calbiochem). The cell membranes were incubated with human erythrocyte GAPDH at 5 U/mL or FLAG-tagged human recombinant wt GAPDH at 5 μg/mL for 2 h at room temperature (RT) and immunoprecipitated by anti-GAPDH-protein G agarose complex or anti-FLAG M2 agarose, respectively. The immunoprecipitates were analyzed by Western blotting.

### Binding of GAPDH to recombinant E-cadherin

A 96-well plate was coated with 100 μL human recombinant E-cadherin Fc chimera (648-EC-100, R&D Systems) /well at 1.5 μg/mL in PBS at 37°C for 1 h. The plate was washed with PBS 4 times and blocked by adding 100 μL/well 1% BSA in PBS at 37°C for 30 min. After washing with PBS 4 times, human erythrocyte GAPDH in PBS was added to the plate at 5 U/mL, and the plate was kept at 37°C for 1 h. The plate was then washed with PBS 4 times and 50 μL of SDS-sample buffer was added to each well. Western blotting was done using 30 μL of the SDS-sample buffer from each well.

### Construction of E-cadherin deletion mutants

Human wild-type E-cadherin and various deletion mutants were constructed as described [35] with some modifications. Total RNA was isolated from MKN-7 cells using the RNeasy Minikit (Qiagen). The cDNA was synthesized using AMV reverse transcriptase (Promega) and a full-length human E-cadherin cDNA fragment containing *Nhe*I and *Xba*I sites was generated by PCR using PfxDNA polymerase (Invitrogen) and sense primer (5’-TATGCTAGCatgggcccttggagc-3’) and antisense primer (5’-TATtctagaaagtcgtcctcgccgcc-3’). The *Nhe*I/*Xba*I fragment of E-cadherin was inserted into a pcDNA3.1/myc-His(-)B vector (Invitrogen). Various extracellular domains of E-cadherin were deleted by inverse PCR using a KOD-Plus-mutagenesis kit (Toyobo) and primer sets as described [35]. The sequence of all expression vectors was confirmed by sequencing. The 293 cell line was transfected with the expression vectors using the Lipofectamine reagent described above and selected with G418. Cell extracts of cell membranes and non-membranes were prepared using ProteoExtract kit.

### Synthesis of Biotinylated MEK Inhibitor I

Biotinylated MEK inhibitor I (b-MEK inh) and acylated MEK inhibitor I (a-MEK inh) were synthesized in our laboratory (S26 Fig.) [36].

### MEK Inhibitor I binding assay

Hs738 cell extracts were prepared using IP buffer (50 mM HEPES-KOH, pH 7.5, 1 M NaCl, 1 mM EDTA, and 2.5 mM EGTA) as described [37]. Streptavidin UltraLink Resin (Pierce) was pre-incubated with b-MEK inh at 2.7 mM for 1 h at RT and then washed with IP buffer. The cell extracts were incubated with the b-MEK inh-treated Streptavidin UltraLink Resin with or without 100 μM MEK inhibitor I or U0126 for 3 h at RT. After 4 washes with IP buffer, the bound proteins were analyzed using SDS-PAGE followed by LC-MS/MS proteomic analysis (LTQ-Orbitrap; Thermo Scientific) or Western blotting.

### Real time RT-PCR

Hs738 cells were cultured for 2 days at 5 x 10^4^ cells/mL in DMEM supplemented with 1% D-FBS and ITH in the presence of MEK inhibitor I. Total RNA was isolated using the RNeasy Minikit (Qiagen). cDNAs were synthesized using AMV reverse transcriptase and random primers (Promega) from 1 μg RNA. Real time RT-PCR was performed on a Thermal Cycler Dice Real Time System (Takara) using SYBR Premix Ex TaqII (Takara). Specific primers were purchased from Takara.

### Exosome preparation

Exosomes were prepared from the cultured supernatant of Hs738 cells as described [38].

### Statistical analysis

All data are representative of at least 3 independent experiments with similar results. Statistical analysis was carried out using Student’s *t*-test.

## Results

### Gastric stromal cells modulate the growth of gastric cancer cells

**Fig 1:**
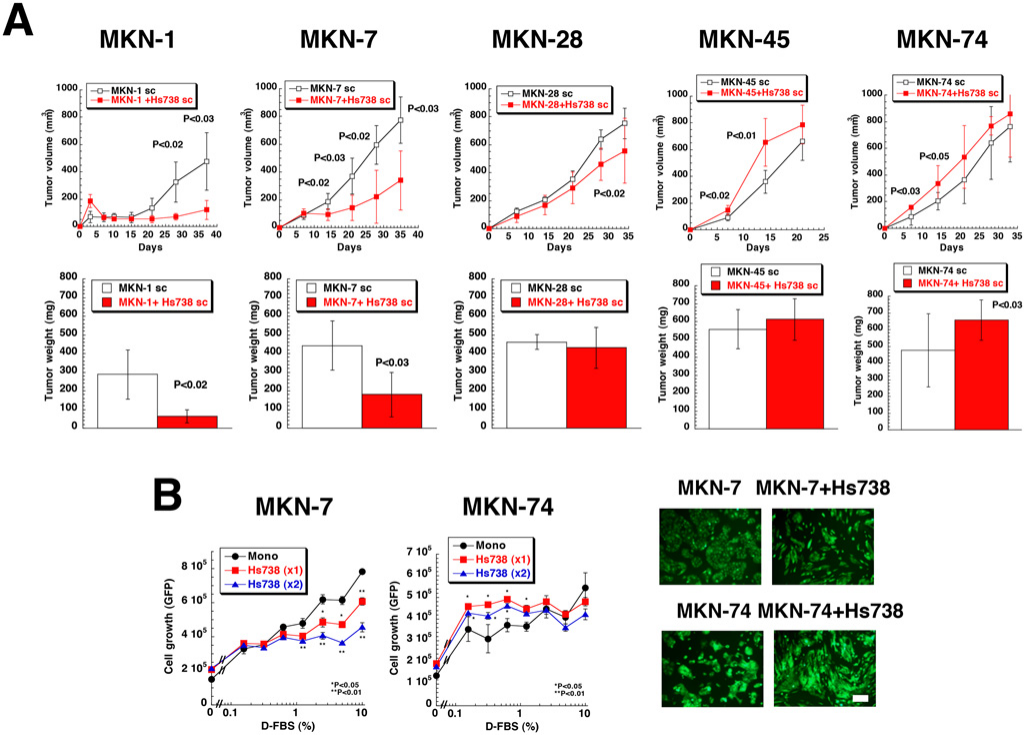
Effect of gastric stromal cells on the growth of gastric cancer cells. (**A**) Gastric cancer cells were injected subcutaneously with or without Hs738 gastric stromal cells in female nude mice. The mice were sacrificed 37, 35, 34, 22, and 33 days after injection of MKN-1, 7, 28, 45, and 74 gastric cancer cells, respectively, and the tumors were excised. The values are means ± s.d. (n = 6). (**B**) MKN-7 and MKN-74 gastric cancer cells were cultured alone (Mono) or co-cultured with Hs738 cells at ratios (gastric cancer: Hs738) 1:1 and 1:2 under the indicated concentrations of D-FBS. The growth of cancer cells was determined measuring GFP fluorescence intensity. The values are means ± s.d. (n = 3). Representative photomicrographs in 1% D-FBS under fluorescence microscopy. Green; GFP. Scale bar is 200 μm.

We established human gastric cancer cell lines that expressed green fluorescent protein (GFP) (S1A Fig.). Setting the same organ, we used human gastric stromal cells (Hs738 cells) that were a mixture of myofibroblasts (vimentin (+) and SM-α-actin (+)) and fibroblasts (vimentin (+) and SM-α-actin (-)) (S1B Fig.) that resembled other stromal cells [4, 24]. All 5 gastric cancer cell lines formed tumors when injected subcutaneously in nude mice (Fig. 1A), but co-injection of Hs738 cells significantly suppressed the tumors of MKN-1, MKN-7 cells, and MKN-28 cells (Fig. 1A). In contrast, the growth of tumors formed by MKN-45 and MKN-74 cells was increased by the presence of Hs738 cells (Fig. 1A). We could not detect metastases of the cancer cells following subcutaneous inoculation, but all cancer cell lines exhibited metastases in the peritoneal cavity when injected into the stomach orthotopically (S2 Fig.). The sizes of the primary tumors initiated by the cancer cell lines injected alone into the stomach did not significantly differ. However, the numbers and frequencies of metastatic foci tended to be higher with MKN-45 and MKN-74 cells (poorly or moderately differentiated cells) and lower in MKN-1, MKN-7 and MKN-28 cells (well-differentiated cell lines [39]) (S2 Fig.). These results showed that the growth of well-differentiated gastric cancer cell lines was suppressed by gastric stromal cells and the growth of undifferentiated gastric cancer cell lines with high metastatic abilities was increased by gastric stromal cells. Since it is suggested there are different mechanisms in the tumor-stromal cell interactions of gastric cancer, we have tried to elucidate them in this study.

To study the tumor-stromal cell interactions, we first developed an *in vitro* co-culture system of gastric cancer cell lines and Hs738 cells as described before [22]. To measure the growth of cancer cells selectively in co-culture with stromal cell, we used GFP-transfected gastric cancer cell lines (S1A Fig.). GFP fluorescence intensities in lysed cells correlated well with cell number as well as MTT assays (S1C Fig.). To reduce the influence of growth factors in FBS, we used dialysed FBS (D-FBS). When the gastric cancer cell lines were co-cultured with Hs738 cells, the growth of MKN-1, MKN-7, and MKN-28 cells was suppressed by the presence of Hs738 cells (Fig. 1B and S3 Fig.). While the growth of MKN-45 cells was not changed by co-culture, that of MKN-74 cells was increased by co-culture with Hs738 cells especially at lower concentrations of D-FBS (Fig. 1B and S3A Fig.). Although the magnitude of influence depended on the concentrations of D-FBS, these results from *in vitro* co-culture experiments were similar to the *in vivo* results (Fig. 1A and S3 Fig.).

### Secreted factors from gastric stromal cells regulate protein synthesis in gastric cancer cells

One of the characteristics of cancer cells is anchorage-independent growth [40]. Because gastric cancer cells were inoculated onto a monolayer of Hs738 cells, the change of growth in co-culture might have been attributable to anchorage-independent growth. When we compared the growth in suspension conditions with normal adherent condition, the growth of all gastric cancer cell lines tended to be suppressed under suspension conditions (S3B Fig.). On the other hand, transwell experiments revealed that the growth of MKN-1, MKN-7, and MKN-28 cells was decreased by co-culture with Hs738 cells, whereas the growth of MKN-74 cells was increased and that of MKN-45 cells was unchanged (S3C Fig.). These results were consistent with *in vitro* co-culture experiments (Fig. 1B and S3A Fig.) and indicated that secreted factors from Hs738 cells instead of anchorage-independent growth were involved in the growth regulation.

**Fig 2:**
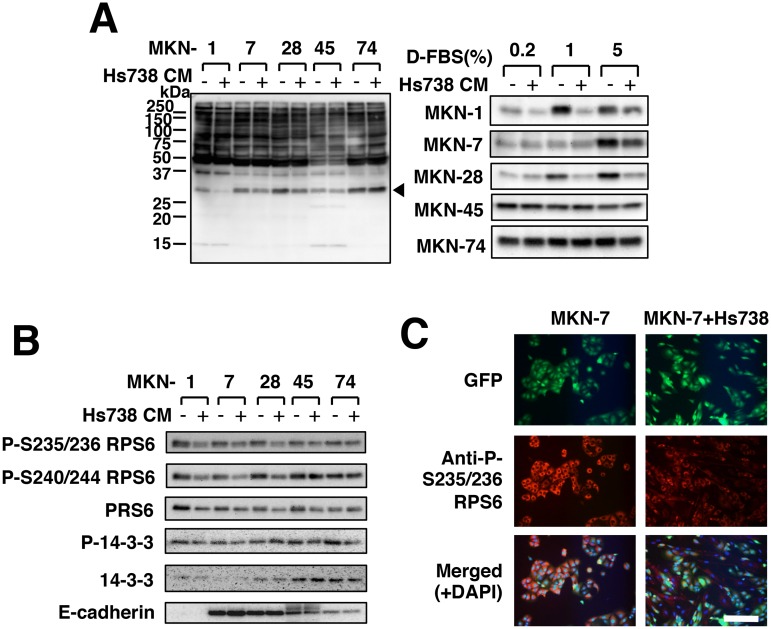
Effect of gastric stromal cells on protein synthesis in gastric cancer cells. (**A**) Gastric cancer cells were cultured for 1 day in Hs738 CM prepared by culturing Hs738 cells with the 1% (left) or indicated concentrations of D-FBS (right) (+) or control medium (-). Ser/Thr-phosphorylated proteins were detected by anti-phospho-Ser/Thr antibody (9614). An arrowhead indicates the position of the band of the right panel. (**B**) The same cell lysates in (A) were assessed with the indicated antibodies. (**C**) MKN-7 cells were cultured with or without Hs738 cells for 1 day. The intracellular phosphorylated RPS6 proteins were immunostained. Scale bar is 200 μm.

Since secreted factors were possibly involved in the growth regulation, we then added conditioned medium (CM) from Hs738 cells to gastric cancer cells and found that phosphorylation of Ser/Thr residues of proteins, especially ~30 kDa proteins, was markedly changed (Fig. 2A). The phosphorylation of ~30 kDa proteins in MKN-1, MKN-7, and MKN-28 cells was significantly decreased by the CM, but that in MKN-45 and MKN-74 cells was not affected (Fig. 2A and S4 Fig.). Proteomic analysis of the ~30 kDa proteins showed that one of the candidates was ribosomal protein S6 (RPS6) (S4B Fig.). siRNAs against RPS6 decreased the phosphorylation of the ~30 kDa proteins as well as phosphorylated RPS6 (S4C Fig.). Furthermore, immunoprecipitates generated by anti-RPS6 antibody were detected by anti-phospho-Ser/Thr antibody as well as anti-phosphorylated RPS6 antibody (S4C Fig.). Thus, the ~30 kDa protein was indeed RPS6. Actually Hs738 CM decreased the phosphorylation of RPS6 in MKN-1, MKN-7, and MKN-28 cells, but not that of 14–3–3 protein, another candidate of the phosphorylated protein (Fig. 2B and S4B Fig.). Furthermore, phosphorylated RPS6 in MKN-7 cells was found to decrease when co-cultured with Hs738 cells (Fig. 2C). Phosphorylation of RPS6 is well known to play a critical role in the regulation of protein synthesis [41]. Thus, our results indicated that Hs738 cells at least downregulated the protein synthesis and suppressed the growth of MKN-1, MKN-7, and MKN-28 cells through secreted factors.

### MEK Inhibitor I modulates tumor-stromal cell interactions

**Fig 3:**
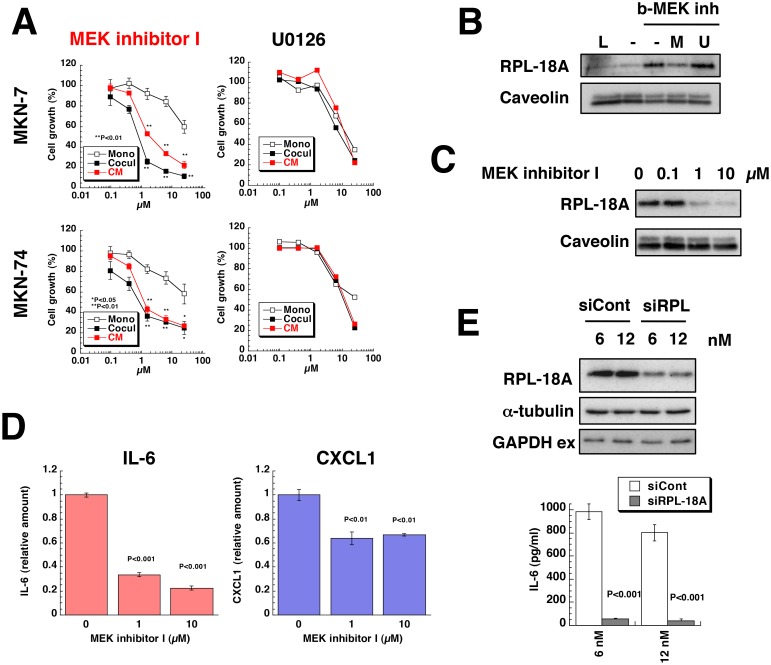
Effect of MEK inhibitor I on co-culture of gastric cancer cells with gastric stromal cells. (**A**) MKN-7 and MKN-74 cells were cultured alone (Mono) or co-cultured with Hs738 (Cocul) for 3 days in the presence of MEK inhibitor I or U0126. Hs738 cells were cultured with the inhibitors for 2 days and CM was prepared. Both gastric cancer lines were cultured in the CM for 3 days. Cell growth was determined by measuring GFP fluorescence intensity. The values are means ± s.d. (n = 3). Cell growth is expressed as a percentage of the value without test compounds in each culture condition. (**B**) Hs738 cell extracts were incubated with b-MEK inh-pretreated streptavidin resin in the presence or absence of MEK inhibitor I (M) or U0126 (U) and the bound proteins were analyzed by Western blotting. L, 1/50 of loaded cell extracts. (**C**) Hs738 cells were cultured with MEK inhibitor I for 2 days and the cell lysates were analyzed by Western blotting. (**D**) Hs738 cells were cultured with MEK inhibitor I for 2 days and the concentrations of IL-6 and CXCL1 in the CM were determined. The values are means ± s.d. (n = 3). (**E**) Hs738 cells were treated with siRNA specific for RPL-18A (siRPL) or negative control (siCont) for 2 days and then re-inoculated followed by further culture for 2 days. The cell lysates were analyzed by Western blotting and the amounts of IL-6 in the CM were determined. The values are means ± s.d. (n = 3).

To identify factors that regulate the growth of gastric cancer cells, we used various small molecule inhibitors, because we previously revealed the critical involvement of IGF-I in tumor-stromal cell interactions of prostate cancer using the same approach [19]. We examined the effects of about 300 inhibitors on the growth of gastric cancer cells with or without Hs738 cells and as a result we focused on MEK inhibitor I (S5 Fig.). MEK inhibitor I, unlike other MEK inhibitors, strongly inhibited the growth of gastric cancer cells when co-cultured with Hs738 cells compared to that of gastric cancer cells cultured alone (Fig. 3A and S6A Fig.). Furthermore, CM prepared from Hs738 cells cultured with MEK inhibitor I also inhibited the growth of gastric cancer cells to the same extent observed in co-culture conditions (Fig. 3A). However, MEK inhibitor I did not show the same enhanced growth-inhibitory effect when the inhibitor was merely added to Hs738 CM (S6B Fig.) suggesting that MEK inhibitor I affected secreted factors from Hs738 cells.

Unexpectedly U0126, a well-known inhibitor of MEK, did not show the same results as MEK inhibitor I (Fig. 3A and S6A Fig.). Thus, it is suggested that MEK inhibitor I could act on other targets. To identify its target, we synthesized a biotinylated MEK inhibitor I (b-MEK inh) (S7 Fig.) and examined its binding proteins from Hs738 cells by proteomic analysis (S8 Fig.). We found that b-MEK inh bound to RPL-18A, 60S ribosomal protein L18a, and excess amounts of MEK inhibitor I, but not U0126, inhibited the binding of b-MEK to RPL-18A (Fig. 3B). Furthermore, MEK inhibitor I treatment resulted in decrease of RPL-18A proteins in Hs738 cells without affecting mRNA expression for RPL-18A (Fig. 3C and S9A Fig.) suggesting that MEK inhibitor I bound to RPL-18A and destabilized it. Thus, these results showed that MEK inhibitor I had a different target such as RPL-18A other than MEK like U0126 and modified secreted factors from Hs738 cells.

### Stromal cells secrete IL-6 to stimulate cancer cells

Since some secreted factors from Hs738 cells were suggested to be affected by MEK inhibitor I (Fig. 3A), we examined the effect of CM on gastric cancer cells. As a result, we found that p70S6 kinase (p70S6K) and RPS6 were downregulated by the CM from MEK inhibitor I-treated Hs738 cells (S10 Fig.). Furthermore, we found that STAT3 was activated by Hs738 CM, but the activation was inhibited in Hs738 CM pretreated with MEK inhibitor I (S10 Fig.). These results suggested that Hs738 cells secreted growth factors and/or cytokines into the CM. Pursuing that hypothesis, we found that Hs738 cells secreted IL-6 and CXCL1 (S11 Fig.) and MEK inhibitor I inhibited release of those factors (Fig. 3D and S11 Fig.). Furthermore, siRNA for RPL-18A, one of targets of MEK inhibitor I, successfully inhibited IL-6 secretion from Hs738 cells (Fig. 3E). Thus, these results indicated that Hs738 cells secreted IL-6 through RPL-18A-dependent mechanism.

**Fig 4:**
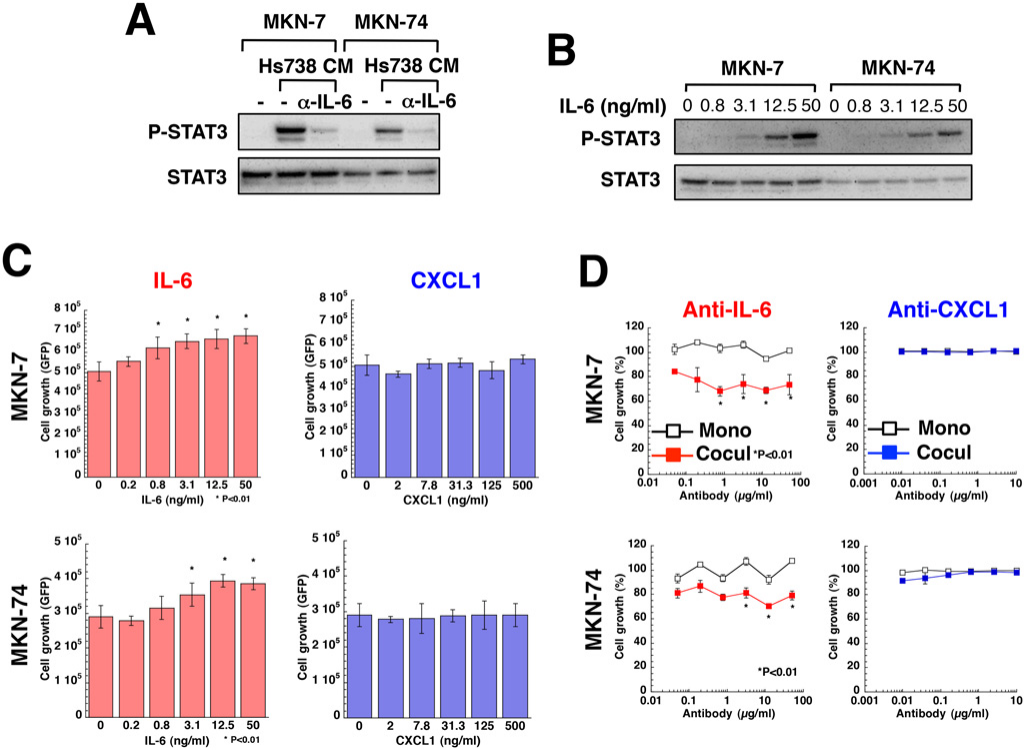
Effect of IL-6 on the growth of gastric cancer cells. (**A**) MKN-7 and MKN-74 cells were cultured for 15 min with or without 50 μg/mL anti-IL-6 neutralizing antibody in Hs738 CM prepared by 2 days of culture. The activation of STAT3 was analyzed by Western blotting. (**B**) MKN-7 and MKN-74 cells were cultured for 15 min with IL-6. The activation of STAT3 was analyzed by Western blotting. (**C**) MKN-7 and MKN-74 cells were cultured with IL-6 or CXCL1 for 3 days. Cell growth was determined by measuring GFP fluorescence intensity. The values are means ± s.d. (n = 3). (**D**) MKN-7 and MKN-74 cells were cultured alone (Mono) or co-cultured with Hs738 cells (Cocul) with the indicated antibodies for 3 days. Cell growth was determined by measuring GFP fluorescence intensity. The values are means ± s.d. (n = 3). Cell growth is expressed as a percentage of the value without antibodies in each culture condition.

Hs738 cells secreted IL-6 and CXCL1 more than did gastric cancer cell lines with the exception of MKN-1. Conversely, gastric cancer cell lines secreted IL-6 soluble receptor, IL-6sR (S12A Fig.). Because IL-6 activates STAT3, the activation of STAT3 by Hs738 CM was inhibited by the addition of IL-6-neutralizing antibody showing the functional involvement of IL-6 in Hs738 CM (Fig. 4A). Moreover, IL-6 activated STAT3 in gastric cancer cells in a dose-dependent fashion (Fig. 4B and S13A Fig.). Actually IL-6 increased the growth of all the gastric cancer cell lines except MKN-45; in contrast, CXCL1 did not affect their growth (Fig. 4C and S13B Fig.). Anti-IL-6 neutralizing antibody did not affect the growth of any of the gastric cancer cells cultured alone, but it inhibited the growth of MKN-7, MKN-28, and MKN-74 cells co-cultured with Hs738 cells (Fig. 4D and S13B Fig.). In contrast, anti-CXCL1 neutralizing antibody did not show such activity (Fig. 4D and S13B Fig.). Although the growth of MKN-1 cells co-cultured with Hs738 was not affected by anti-IL-6 antibody, this was likely due to the cells’ autocrine secretion of IL-6 (S12A Fig. and S13B). With regard to MKN-45 cells, growth was unaffected by addition of IL-6 or anti-IL-6 antibody. Whereas IL-6 did not affect the growth of 3 other signet ring cell type gastric cancer cell lines, it activated STAT3 in all the cell lines as it did in MKN-45 cells (S13A Fig. and S14). Since IL-6 reportedly acts as a survival signal as well as a growth signal for gastric cancer cells [42, 43], Hs738 cells possibly stimulates the growth or survival of gastric cancer cells through IL-6.

### Cancer cells secrete PGE2 and TNFα to stimulate IL-6 production from stromal cells

**Fig 5:**
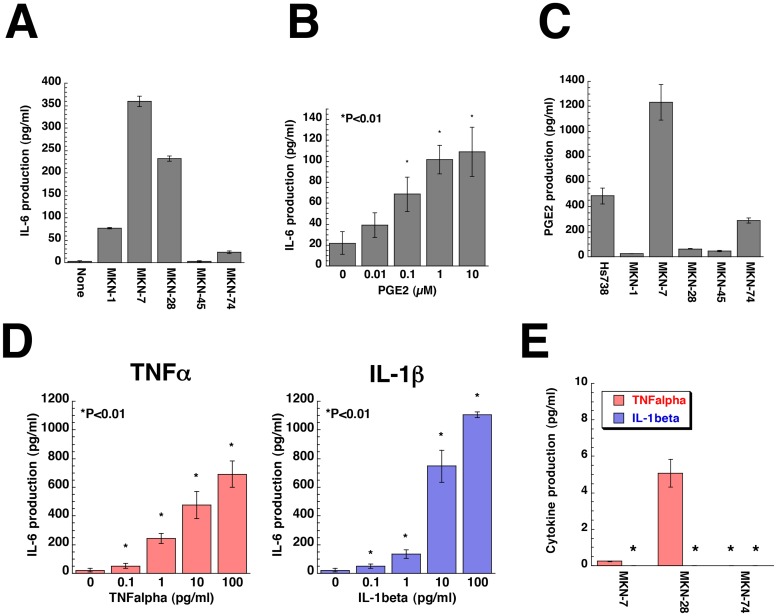
Gastric cancer cells secrete PGE2 and TNFα to stimulate IL-6 production by gastric stromal cells. (**A**) Hs738 cells were cultured for 1 day in CM prepared from the indicated cells. The amount of IL-6 in the cultured supernatant was determined. The values are means ± s.d. (n = 3). (**B**) Hs738 cells were cultured with PGE2 for 1 day. The amount of IL-6 in the cultured supernatant was determined. The values are means ± s.d. (n = 3). (**C**) The amount of PGE2 in CM prepared from the indicated cells cultured for 2 days was determined. The values are means ± s.d. (n = 3). (**D**) Hs738 cells were cultured with TNFα or IL-1β for 1 day. The amount of IL-6 in CM was determined. The values are means ± s.d. (n = 3). (**E**) The amounts of TNFα and IL-1β in CM prepared from the indicated cells cultured for 2 days were determined after the concentration of the CM. The values are means ± s.d. (n = 3). *Under detectable level.

Cancer cells are known to respond to secreted factors from stromal cells through production of factors such as TGF-β [4, 44, 45]. We examined the effect of gastric cancer cell CM on IL-6 secretion from Hs738 cells. As a result, we found that CM from MKN-7, MKN-28, and MKN-74 cells increased IL-6 secretion from Hs738 cells (Fig. 5A). Prostanoids and Wnt signaling are known to be involved in progression of gastric cancer [46, 47]. Thus, we examined the effect of various prostanoids on IL-6 secretion and found that PGE2 increased IL-6 secretion from Hs738 cells in a dose-dependent fashion (Fig. 5B and S12B Fig.). We next examined prostanoids in the CM and found that MKN-7 and MKN-74 cells secreted PGE2 (Fig. 5C and S12C Fig.). We previously reported that TNFα and IL-1β stimulated stromal cells to secrete IL-6 [48]. As expected, TNFα and IL-1β increased IL-6 secretion from Hs738 cells at very low concentrations (Fig. 5D). When CM from MKN-7, MKN-28 and MKN-74 cells was concentrated, TNFα, but not IL-1β was detected in MKN-28 CM at concentrations that were sufficient to stimulate Hs738 cells to secrete IL-6 (Fig. 5E). In case of MKN-7 CM the amounts of PGE2 and TNFα are less sufficient for stimulation of IL-6 secretion. There is a possibility that the synergistic effect of PGE2 and TNFα and/or additional factor(s) might be involved in the IL-6 stimulation. Thus, some of the gastric cancer cells secreted PGE2 and TNFα stimulating the stromal cells to secrete IL-6 that supported the growth of cancer cells (S25 Fig.).

### Stromal cells secrete GAPDH to inhibit cancer cells

**Fig 6:**
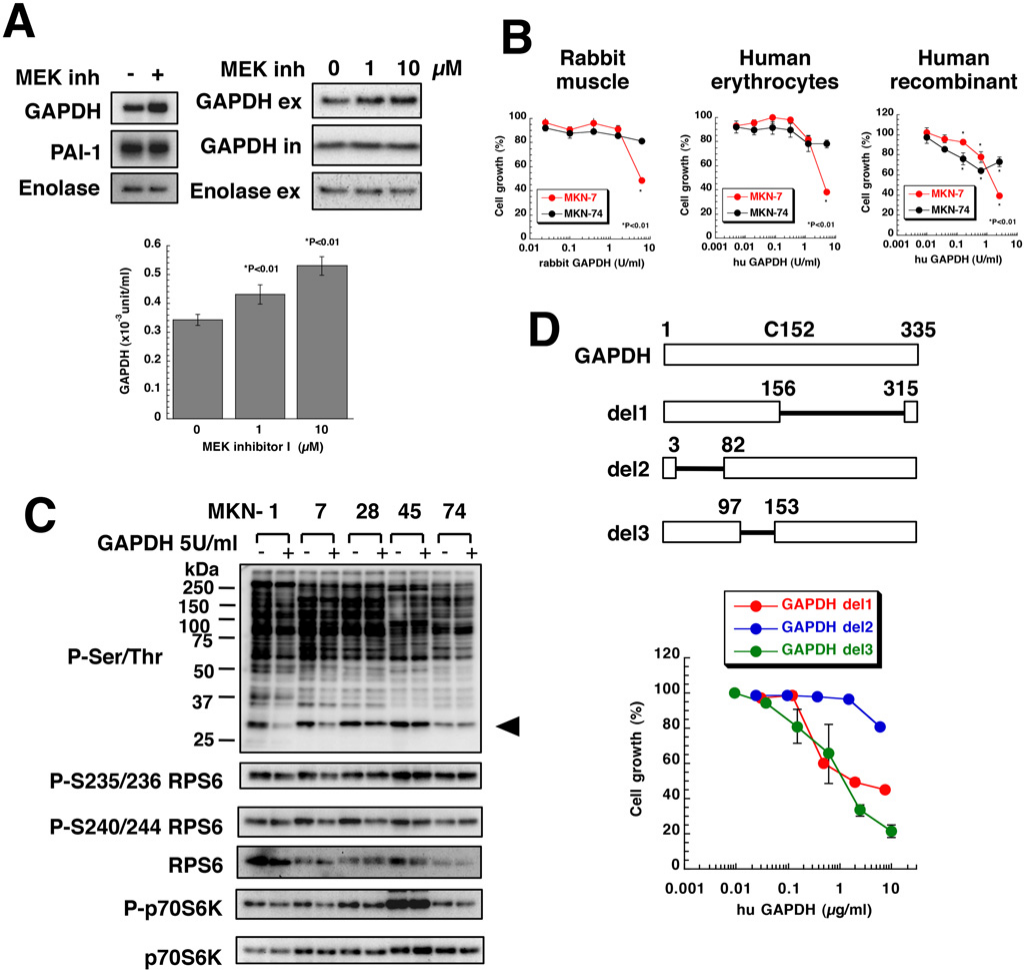
Gastric stromal cells secreted GAPDH and suppressed the growth of gastric cancer cells. (**A**) Concentrated Hs738 CM prepared by culturing with or without 10 μM MEK inhibitor I was analyzed by Western blot (upper left). Hs738 cells were cultured with MEK inhibitor I for 2 days and the cultured supernatant was collected. GAPDH and enolase in the culture supernatant (ex) and the cell lysates (in) was analyzed by Western blot (upper right). GAPDH enzyme activity was examined in the cultured supernatant (lower). The values are means ± s.d. (n = 3). (**B**) MKN-7 and MKN-74 cells were cultured with various amounts of GAPDH for 3 days. Cell growth was determined using MTT. The values are means ± s.d. (n = 3). (**C**) Gastric cancer cells were cultured with or without human erythrocyte GAPDH at 5 U/mL for 1 day. The cell lysates were analyzed by Western blotting with anti-phospho-Ser/Thr antibody (9624) and the indicated antibodies. An arrowhead indicates the position of RPS6. (**D**) MKN-7 cells were cultured with human recombinant deletion mutants of GAPDH for 3 days. Cell growth was determined using MTT. The values are means ± s.d. (n = 3). Cell growth is expressed as a percentage of the value without GAPDH in each culture condition.

Hs738 CM downregulated phosphorylation of RPS6 and this inhibitory effect was more pronounced in MEK inhibitor I-treated CM suggesting that Hs738 CM contained growth-inhibitory activities (Fig. 2, S3C Fig., and S15 Fig.). To analyze those factors, we concentrated CM from Hs738 cells pretreated with MEK inhibitor I and purified the proteins (S15 Fig.). Proteomic analysis of gel filtrated fractions revealed that the candidate proteins were enolase, PAI-1, and GAPDH (S15 Fig.). Western blotting revealed that one of the growth inhibitory factors might be GAPDH. In fact, MEK inhibitor I increased extracellular secretion of GAPDH without affecting the intracellular levels of GAPDH and the extracellular enolase (Fig. 6A). The amount of secreted GAPDH was higher in Hs738 cells than gastric cancer cell lines (S16 Fig.). Furthermore, other stromal cells also secreted GAPDH (S16 Fig.). Although GAPDH is reported to be secreted and to change cell morphology [34], its growth inhibitory activity has not been reported.

GAPDH from rabbit muscle as well as human erythrocytes and human recombinant GAPDH were found to strongly inhibit the growth of MKN-7 cells, whereas MKN-74 cells were weakly inhibited (Fig. 6B). We also found that GAPDH inhibited the growth of several cancer cell lines (S17 Fig.). In contrast, GAPDH did not affect the growth of various stromal cells (S17 Fig.). Furthermore, we found that GAPDH decreased phosphorylation of RPS6 and its upstream regulator, p70S6K, in MKN-1, MKN-7, and MKN-28 cells (Fig. 6C) as did Hs738 CM (Fig. 2A and S4A Fig.). Thus, it is considered that GAPDH inhibited protein synthesis and growth of MKN-1, MKN-7, MKN-28 cells.

We next examined whether the growth inhibitory effect of GAPDH required its enzymatic activity. FLAG-tagged mutant GAPDH (C152S) had no enzyme activity (S18A Fig.), but it still significantly inhibited the growth of MKN-7 cells (S18A Fig.). To determine whether the catalytic domain was indispensable for the growth inhibitory activity, we constructed several deletion mutants of GAPDH (S18B Fig.). As a result, we found that GAPDH del1 lacking the C-terminal domain of GAPDH inhibited the growth of MKN-7 cells (Fig. 6D). While GAPDH del3 lacking 98–152 amino acids in the N-terminal domain also strongly inhibited the growth, GAPDH del2 lacking 4–81 amino acids in N-terminal domain failed to inhibit the growth (Fig. 6D). These results clearly indicated that the N-terminal domain especially 4–81 amino acids without the catalytic domain was essential for the growth inhibitory activity.

### Extracellular GAPDH binds to E-cadherin

**Fig 7:**
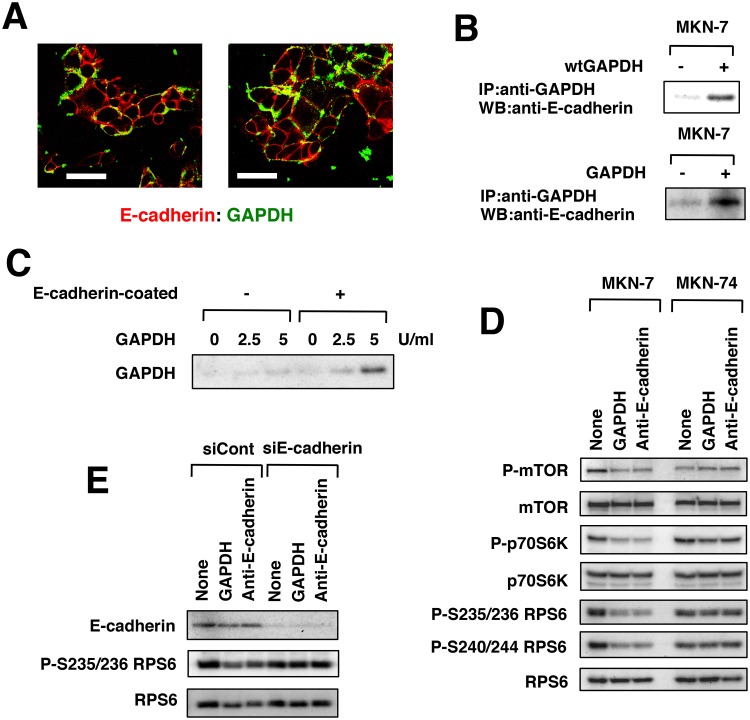
Extracellular (secreted) GAPDH bound to E-cadherin. (**A**) MKN-7 cells were cultured with human erythrocyte GAPDH at 5 U/mL for 1 day. The cells were fixed under nonpermeabilizing conditions, stained with the indicated antibodies and analyzed by confocal microscopy. Scale bar is 50 μm. (**B**) Cell membranes of MKN-7 cells were incubated with FLAG-tagged human recombinant wtGAPDH or human erythrocyte GAPDH. The immunoprecipitates generated with the indicated antibodies were analyzed by Western blotting with anti-E-cadherin antibody. (**C**) Human erythrocyte GAPDH was added to a human recombinant E-cadherin-coated plate. GAPDH bound to the plate was detected by Western blot. (**D**) MKN-7 and MKN-74 cells were cultured with human erythrocyte GAPDH at 5 U/mL or anti-E-cadherin antibody at 1 μg/mL for 1 day. The mTOR-p70S6K pathway was analyzed by Western blot. (**E**) MKN-7 cells were transfected with control siRNA or E-cadherin siRNA for 3 days and then further cultured with human erythrocyte GAPDH at 5 U/mL or anti-E-cadherin antibody at 1 μg/mL for 1 day. E-cadherin and phosphorylated RPS6 were analyzed by Western blotting.

Immunofluorescence with anti-GAPDH antibody (in the absence of permeabilizing conditions) suggested that GAPDH bound to cell membranes, especially to cell-to-cell junctions (S19B Fig.). The sites at which GAPDH bound were similar to the sites of E-cadherin expression (S19B Fig.). Confocal laser scanning microscopy revealed that GAPDH was partially colocalized with E-cadherin in MKN-7 cells (Fig. 7A). We then used cell membranes and found that GAPDH as well as FLAG-tagged GAPDH directly bound to E-cadherin (Fig. 7B and S20 Fig.). Furthermore, GAPDH bound to E-cadherin-coated plates *in vitro* (Fig. 7C). GAPDH inhibited mTOR activation and its downstream p70S6K activation in MKN-7 cells, but not in MKN-74 cells (Fig. 7D and S21 Fig.). This result was consistent with GAPDH sensitivity (Fig. 6B). Anti-E-cadherin antibody inhibited the activation of the mTOR-p70S6K pathway in MKN-7 cells (Fig. 7D), but neither GAPDH nor anti-E-cadherin antibody inhibited possible regulators of the mTOR-p70S6K pathway, including Src, Akt, MAPK, AMPK and β-catenin (S21 Fig.). However, E-cadherin siRNA attenuated the downregulation of RPS6 by GAPDH as well as anti-E-cadherin antibody (Fig. 7E).

Thus, the growth-inhibitory activity of GAPDH was correlated with downregulation of the mTOR-p70S6K pathway but the mechanism by which the interference of E-cadherin function led to the down-regulation of the mTOR-p70S6K pathway is unknown. MKN-45 and MKN-74 cells expressed E-cadherin weakly compared to other gastric cancer cells and did not respond to the growth inhibitory effect of GAPDH (Fig. 2B and S17 Fig.). Although E-cadherin-null MKN-1 cells were exceptional, these results showed that GAPDH interfered with E-cadherin function and suppressed protein synthesis and growth of MKN-7 and MKN-28 cells.

## Discussion

Stromal cells regulate the growth of cancer cells positively and negatively. In this study we have found that Hs738 gastric stromal cells secrete IL-6 and GAPDH for positive and negative regulation of cancer cells, respectively. While IL-6 stimulated the growth and survival of almost all gastric cancer cell lines used here, GAPDH suppressed the growth of MKN-7 and MKN-28 cells. Overall it is considered that Hs738 cells suppressed the growth of MKN-7 and MKN-28 cells through GAPDH, but they stimulated that of MKN-45 and MKN-74 cells through IL-6. MKN-45 and MKN-74 cells (undifferentiated cell lines) showed high metastatic ability *in vivo* (S2 Fig.). They might induce the epithelial-mesenchymal transition to obtain the high metastatic ability, because they expressed E-cadherin at low levels and failed to respond to GAPDH. Thus, stromal cells could not suppress those cells.

When we examined STAT3 activation in human gastric cancer tissues, activation was detected in more than half of the adenocarcinoma tissues (S22 Fig.), consistent with other reports [49, 50]. Although STAT3 activation was not detected in GIST tissues, it was detected in half of the signet ring cell carcinoma tissues (S22 Fig.). These results indicated that STAT3 activation by IL-6 is involved in clinical cancer. It is reported that IL-6 also plays an important role in other cancer [51]. Since IL-6 is secreted from stromal cells derived from other organs [48], IL-6 is one of general factors that stimulate cancer development. Indeed, during preparation of our manuscript, Kinoshita et al has excellently proved that IL-6 secreted from gastric stromal cells promotes gastric tumorigenesis [52]. Although the experimental approaches are different, our present findings strongly support their findings.

Gastric cancer cells secreted at least PGE2 and TNFα to stimulate IL-6 production by stromal cells. However, the CM of gastric cancer cells did not affect GAPDH secretion from Hs738 cells (S16 Fig.). Thus, the PGE2-TNFα-IL-6 pathway is a paracrine loop for gastric cancer progression. *Helicobacter pylori* infection is the major cause of gastric cancer and *H*. *pylori* not only directly activates oncogenic pathways of epithelial cells, but also induces chronic inflammation [53]. Since inflammatory reactions are mediated by inflammatory cytokines such as PGE2, TNFα, and IL-6, our study could provide an explanation for gastric carcinogenesis. Furthermore, since the PGE2-TNFα-IL-6 pathway is considered to likely occur in other cancer, the interference of such positive regulation is a reasonable target for anti-cancer strategies.

Because GAPDH is one of abundant housekeeping proteins in a cell, it is unexpected that extracellular GAPDH secreted from a cell has different functions. Yamaji et al reported that GAPDH is secreted from some cancer cells and inhibits cell spreading [34]. However, the growth inhibitory activity of GAPDH against cancer cells has not been reported. This is due to the sensitivity of cancer cells to GAPDH. As shown in S17 Fig., only some cancer cell lines, not all, responded to the growth inhibitory activity. By constructing several mutants of GAPDH, we have found that the N-terminal domain of GAPDH was essential to exert the growth inhibitory activity (Fig. 6D). It is interesting that the growth inhibitory activity does not need the catalytic domain for original enzyme activity. Unexpectedly, the N-terminal peptide of GAPDH has been recently reported to have antifungal activity against *Candida albicans* through internalization [54]. However, immunofluorescence with anti-FLAG antibody under cell-permeabilized conditions revealed that GAPDH was not incorporated into MKN-7 cells (S19A Fig.). Thus, the mechanism of its action is not considered to be the same.

GAPDH is found to bind to E-cadherin and to downregulate mTOR-p70S6K pathway. Anti-E-cadherin antibody also downregualted mTOR-p70S6K pathway suggesting the obstruction of extracellular regions of E-cadherin results in the downregulation of mTOR-p70S6K pathway. E-cadherin has 5 repeated extracellular domains (ECs), and EC1- EC4 are involved in its homophillic adhesion [35]. We constructed 293 cells expressing various deletion mutants of E-cadherin (S23 Fig.) and determined their binding activity to GAPDH. We found that GAPDH bound to deletion mutants of EC1, EC2, and EC3 to the same extent as did wild-type E-cadherin. In contrast, it bound with great efficiency to deletion mutants of EC4 and EC5 (S23 Fig.), indicating that binding of GAPDH to E-cadherin was not dependent on the homophillic adhesion activity of E-cadherin. Since various stromal cells secrete GAPDH (S16 Fig.), this negative regulation likely occurs in any organs. Thus, the mechanism by which GAPDH inhibits mTOR-p70S6K pathway through E-cadherin is still unknown, but GAPDH is newly rediscovered as an anti-cancer strategy.

We used MEK inhibitor I as a tool to elucidate the tumor-stromal cell interactions of gastric cancer cells. Although MEK inhibitor I was originally created as an inhibitor of MEK, we found its new target, RPL-18A. The siRNA for RPL-18A successfully inhibited the secretion of IL-6 from Hs738 cells like MEK inhibitor I treatment (Fig. 3E). However, it failed to increase extracellular GAPDH (Fig. 3E) unlike MEK inhibitor I. Exosomes reportedly play a critical role in delivering many intracellular molecules such as miRNA [38]. However, MEK inhibitor I did not increase GAPDH secretion by exosomes (S9B Fig.). These results indicate that MEK inhibitor I acted on at least RPL-18A to modulate tumor-stromal cell interactions, but it still has another target in respect to GAPDH secretion. We previously reported that phthoxazolin A, a natural compound, decreased the expression of SM-α-actin and IGF-I in PrSC human prostate stromal cells [24]. It also suppressed the growth of human prostate cancer cells in co-culture. However, MEK inhibitor I did not affect the expression of vimentin or SM-α-actin in Hs738 cells nor inhibit the growth of Hs738 cells (S24 Fig.) suggesting that the action of MEK inhibitor I is different from that of phthoxazolin A.

Taken together, our results showed that gastric stromal cells positively regulated the growth of gastric cancer cells through the PGE2-TNFα-IL-6 paracrine pathway, but negatively through secreted GAPDH that bound to E-cadherin (S25 Fig.). The balance between those signals and the sensitivity of cancer cells to them determines the overall outcome. That is, cancer cells differ in their sensitivities to IL-6 and also GAPDH. Although the precise mechanism by which stromal cells secrete GAPDH remains to be elucidated, compounds that augment the secretion could be new candidates for antitumor drugs. Thus, our results suggest that targeting stromal cells, especially the negative regulation of cancer cells, is a feasible new anti-cancer strategy.

## Supporting Information

S1 Fig GFP-transfected gastric cancer cells and Hs738 gastric stromal cells. (**A**) Gastric cancer cell lines were stably transfected with a GFP expression vector. Photos were taken under phase contrast microscopy (left) and fluorescence microscopy (right). Scale bar is 200 μm. (**B**) Characteristics of Hs738 cells. Hs738 human gastric stromal cells were a mixture of fibroblasts expressing vimentin without SM-α-actin and myofibroblasts expressing both vimentin and SM-α-actin. A photo of Hs738 cells in upper left was taken under phase contrast microscopy. Western blots in upper right show that Hs738 cells express vimentin and SM-α-actin compared with MKN-74 gastric cancer cells expressing only cytokeratin. Lower panels show immunofluorescence staining of Hs738 cells with vimentin (green), SM-α-actin (red), and also DAPI staining (blue). Scale bar is 200 μm. (**C**) Cell growth of GFP-transfected cells. GFP-transfected gastric cancer cell lines and untransfected DU-145 prostate cancer cells were inoculated in 96-well plates with 10% FBS at the indicated numbers per well. After overnight incubation, the cell numbers were determined using MTT (left) or measuring GFP fluorescence intensity (right). Cell numbers correlated well with GFP fluorescence intensity as well as MTT in gastric cancer cell lines, but not in DU-145 cells without GFP transfection. The values are means ± s.d. (n = 3).
(PDF)

S2 Fig Orthotopic implantation of gastric cancer cells *in vivo*. Gastric cancer cells were inoculated orthotopically into the stomach of female nude mice. The mice were sacrificed on days 37, 35, 34, 22, and 33 after the inoculation of MKN-1, 7, 28, 45, and 74 cells, respectively. The number of metastatic foci in the peritoneal cavity was counted under fluorescence microscopy (upper right) and the primary tumor was excised (upper left). Tumor size was classified as >3mm, 1.5–3 mm, and <1.5 mm. Numbers indicate animals positive for metastases. Representative photos of the peritoneal cavity are shown (lower panels). There are GFP-positive primary tumors and metastatic foci. The values are means ± s.d. (n = 6).
(PDF)

S3 Fig Growth of gastric cancer cells on various culture conditions. (**A**) Co-culture of gastric cancer cells and Hs738 cells. Gastric cancer cells were cultured alone (Mono) or co-cultured with Hs738 cells at a ratio (gastric cancer:Hs738) of 1:1 in the indicated concentrations of D-FBS. The growth of cancer cells was determined measuring GFP fluorescence intensity. The values are means ± s.d. (n = 3). (**B**) Gastric cancer cells were cultured in a suspension culture plate (Suspension) or a normal culture plate (Solid) for 3 days. The growth of cancer cells was determined using MTT. The values are means ± s.d. (n = 3). (**C**) In transwell plates, gastric cancer cells (inner wells) were cultured alone (Transwell Mono) or co-cultured with Hs738 cells (outer wells) (Transwell Cocul) for 3 days. The growth of cancer cells was determined measuring GFP fluorescence intensity. The values are means ± s.d. (n = 3).
(PDF)

S4 Fig Effect of Hs738 CM on phosphorylated proteins in gastric cancer cells. (**A**) Gastric cancer cells were cultured in the conditioned medium of Hs738 cells prepared by culturing Hs738 cells with 1% (left) or the indicated concentrations of D-FBS (right) (+) or control medium (-). Ser/Thr-phosphorylated proteins were detected by anti-phospho-Ser/Thr antibody (9624). An arrowhead indicates the position of the band of the right panel. (**B**) Partial purification of phosphorylated proteins in MKN-7 cells. Phosphorylated proteins were partially purified using an anionic column and detected by anti-phospho-Ser/Thr antibody (9624). The bands indicated by an arrowhead in lanes 4 and 5 were excised and analyzed by LC-MS/MS. The bands were deduced to be 14–3–3 protein epsilon and ribosomal protein S6 (RPS6). (**C**) Effect of siRNA against RPS6 on MNK-7 cells. MKN-7 cells were transfected with siRNAs against RPS6 (si#1 and si#2) for 2 days and the indicated proteins were analyzed by Western blot (left). MKN-7 cell lysates were immunoprecipitated with anti-RPS6 or normal Ig and the immunoprecipitates were detected by the indicated antibodies (right).
(PDF)

S5 Fig Effect of various inhibitors on co-culture. MKN-7 and MKN-74 cells were cultured with or without Hs738 cells for 3 days in the presence of various inhibitors (about 300 compound; for details, http://scads.jfcr.or.jp/kit/kit.html) at 1 and 10 μM. The growth of cancer cells was expressed as a heat map. An arrow indicates the position of MEK inhibitor I.
(PDF)

S6 Fig Effect of various MEK inhibitors on co-culture. (**A**) GFP-expressing MKN-7, MKN-74, and DU-145 cells were cultured alone (mo) or co-cultured with Hs738 cells (co) for 3 days in the presence of inhibitors. The cell growth was determined measuring GFP fluorescence intensity. The values are means ± s.d. (n = 3). (**B**) MKN-7 and MKN-74 cells were cultured with MEK inhibitor I for 3 days in the presence or absence of Hs738 CM prepared by culturing Hs738 cells without inhibitors for 2 days. The cell growth was determined measuring GFP fluorescence intensity. The values are means ± s.d. (n = 3). Cell growth is expressed as a percentage of the value without test compounds in each culture condition.
(PDF)

S7 Fig Design of MEK inhibitor I derivatives. Acylated MEK inhibitor I (a-MEK inh) and biotinylated MEK inhibitor I (b-MEK inh) were synthesized. GFP-expressing MKN-7 cells were cultured alone (mo) or co-cultured with Hs738 cells (co) for 3 days in the presence of inhibitors. Cell growth was determined by measuring GFP fluorescence intensity. The values are means ± s.d. (n = 3). Cell growth is expressed as a percentage of the value without test compounds in each culture condition. Comparing the structures of MEK inhibitor I and U0126, amine of aniline moiety was modified. Because a-MEK inh sustained almost the same activity as MEK inhibitor I, we synthesized b-MEK inh by modifying the same site.
(PDF)

S8 Fig Binding assay of b-MEK inh to cell extracts. Hs738 cell extracts were incubated with b-MEK inh-pretreated Streptavidin resin and the bound proteins were analyzed by SDS-PAGE. Coomassie brilliant blue (CBB) staining revealed some bands specific to b-MEK inh (Arrow). M, marker; 1, Hs738 cell extracts; 2, negative control; 3, b-MEK inh-treated. Bands specific to b-MEK inh were analyzed by LC-MS/MS. LC-MS/MS analysis deduced several molecules such as RPL-18A and caveolin as candidates.
(PDF)

S9 Fig Effect of MEK inhibitor I on RPL-18A mRNA and exosome. Hs738 cells were cultured with the indicated concentrations of MEK inhibitor I for 2 days. (**A**) Total RNAs were collected and RPL-18A mRNA levels were analyzed by real time RT-PCR using b-actin as a reference. (**B**) Exosomes were prepared from the cultured supernatant and analyzed by Western blot. CD63 is a marker of the exosome.
(PDF)

S10 Fig Effect of MEK inhibitor I on MKN-7 cells. MKN-7 cells were cultured for the indicated times with or without 10 μM MEK inhibitor I or Hs738 CM prepared by culturing Hs738 cells with or without MEK inhibitor I for 2 days. The cell lysates were analyzed by Western blot with anti-phospho-(Ser/Thr) antibody (9624) and other indicated antibodies. An arrowhead indicates the position of RPS6 protein.
(PDF)

S11 Fig Cytokine antibody array analysis of Hs738 CM. Hs738 cells were cultured with or without 10 μM MEK inhibitor I for 2 days. The cultured supernatants were applied onto human cytokine antibody array (C series 2000; for details, http://www.raybiotech.com). Arrows indicate the positions of IL-6 and CXCL1 duplicated spots. Lower panels are lists of the arrays.
(PDF)

S12 Fig Cytokine production in various cell lines. (**A**) IL-6, IL-6sR, and CXCL1 in CM. Cells were cultured for 2 days and the amounts of IL-6, IL-6sR, and CXCL1 in the CM were determined. The values are means ± s.d. (n = 3). (**B**) Effect of various prostanoids on IL-6 secretion. Hs738 cells were cultured with 10μM various prostanoids for 1 day. The amounts of IL-6 in the cultured supernatant were determined. The values are means ± s.d. (n = 3). (**C**) Prostanoids production in cells. The amounts of PGI_2_, TXA_2_, and leukotriene B4 in CM prepared from the indicated cells cultured for 2 days were determined. The values are means ± s.d. (n = 3).
(PDF)

S13 Fig Effect of IL-6 on gastric cancer cells. (**A**) STAT-3 activation in gastric cancer cells. Cells were cultured for 15 min with 50 ng/ml of IL-6. The activation of STAT3 was analyzed by Western blot. (**B**) Effect of IL-6, CXCL1, anti-IL-6, and anti-CXCL-1 antibodies on growth of gastric cancer cells. Cells were cultured with IL-6, CXCL1, anti-IL-6, or anti-CXCL-1 antibodies (Mono) for 3 days or co-cultured with Hs738 cells (Cocul) for 3 days. Cell growth was determined measuring GFP fluorescence intensity. The values are means ± s.d. (n = 3). For anti-IL-6 and anti-CXCL-1 antibodies, cell growth is expressed as a percentage of the value without antibodies in each culture condition.
(PDF)

S14 Fig Effect of IL-6 on signet ring cell type gastric cancer cells. Cells were cultured with IL-6 for 3 days. Cell growth was determined using MTT. The values are means ± s.d. (n = 3).
(PDF)

S15 Fig Proteomic analysis of Hs738 CM. Hs738 CM prepared by culturing Hs738 cells with or without 10 μM MEK inhibitor I for 3 days was concentrated and separated by gel filtration. MKN-7 cells were cultured with the fractionated CM concentrate for 3 days and the cell growth was determined using MTT (left). The fractions with growth inhibitory activity (green circle) were pooled and applied onto 2D gel electrophoresis (right). Spots, in which expressions in MEK inhibitor I-treated cells were higher than control, were analyzed by LC-MS/MS or MALDI-TOF-MS. LC-MS/MS analysis deduced PAI-precursor protein and α-enolase and MALDI-TOF-MS deduced GAPDH.
(PDF)

S16 Fig GAPDH secretion in various cells. Cells were cultured with or without 10 μM MEK inhibitor I for 2 days (upper). Hs738 cells were cultured for 1 day in CM prepared by culturing gastric cancer cells for 2 days (middle). Various stromal cells were cultured for 2 days (lower). GAPDH in the cultured supernatant was analyzed by Western blot.
(PDF)

S17 Fig Effect of GAPDH on various cells. Cells were cultured with human erythrocyte GAPDH for 3 days. Cell growth was determined using MTT. The values are means ± s.d. (n = 3). Cell growth is expressed as a percentage of the value without hu GAPDH.
(PDF)

S18 Fig Construction of various mutants of recombinant GAPDH. (**A**) Human recombinant wild type (wt) and mutant (mt) GAPDH were detected by Western blot using anti-GAPDH and anti-FLAG antibodies (upper). GAPDH enzyme activity of the recombinant GAPDH was measured. MKN-7 cells were cultured with wt and mt GAPDH for 3 days. Cell growth was determined using MTT. The values are means ± s.d. (n = 3). Cell growth is expressed as a percentage of the value without hu GAPDH. (**B**) Human recombinant wild type (wt) and deletion mutants of GAPDH were constructed using ClearColiBL21. GAPDH del1 lacks the C-terminal domain, GAPDH del2 lacks 4–81 amino acids in the N-terminal domain, and GAPDH del3 lacks 98–152 amino acids in the N-terminal domain. Estimated sizes are 38, 19.3, 27.1, and 30.3 kDa for wt, del1, del2, and del3, respectively. They were separated by SDS-PAGE and assessed by CBB staining (left) and Western blot with anti-FLAG antibody (right).
(PDF)

S19 Fig Immunofluorescence of GAPDH and E-cadherin. (**A**) Effect of recombinant GAPDH on MKN-7 cells. MKN-7 cells were cultured with human recombinant wtGAPDH at 15 μg/ml for 1 day. The cells were fixed under cell-permeabilized conditions, immunostained with anti-FLAG and anti-mouse IgG_1_ Alexa Fluor 546 antibodies, and analyzed by confocal microscopy. Scale bar is 20 μm. (**B**) MKN-7 cells were cultured with human erythrocyte GAPDH at 5 U/ml for 1 day (left). The cells were fixed under nonpermeabilizing conditions and stained with the indicated antibodies. Scale bars are 50 μm.
(PDF)

S20 Fig Binding of GAPDH to E-cadherin. Cell membranes of MKN-74 cells were incubated with FLAG-tagged human recombinant wild type GAPDH or human erythrocyte GAPDH. The immunoprecipitates generated by the indicated antibodies were analyzed by Western blot with anti-E-cadherin antibody. No band was detected by anti-integrin β1 antibody.
(PDF)

S21 Fig Effect of GAPDH on various cell signal molecules. MKN-7 cells were cultured with human erythrocyte GAPDH at 5 U/ml or recombinant wild type (wt) GAPDH at 5 μg/ml for the indicated times. mTOR-p70S6K pathway was analyzed by Western blot (left). MKN-7 cells were cultured with human erythrocyte GAPDH at 5 U/ml or anti-E-cadherin antibody at 1 μg/ml for 30 min. Activated forms of the indicated proteins and b-catenin were analyzed by Western blot (right).
(PDF)

S22 Fig STAT3 activation in human gastric cancer tissues. Tissue sections of human gastric cancer and GIST were stained with anti-phospho-STAT3. Numbers in parentheses are percent. Representative photos of the immunostained sections are shown.
(PDF)

S23 Fig Construction of mutant E-cadherin and binding assay to GAPDH. (**A**) 293 cells expressing Myc-tagged wild-type E-cadherin or its various extracellular domain deletion mutants as illustrated above were established. Non-membrane (N) and membrane (M) extracts were analyzed by Western blot. All cells expressed E-cadherin or its mutants on membranes. E-cadherin has five extracellular repeats (EC1-EC5). The numbers above the columns indicate the amino acid number, counting from the start of the coding region. S. signal peptide; P, propeptide; TM, transmembrane domain; C, cytoplasmic domain. The illustration was modified from ref 35. (**B**) Cell membranes of MKN-7 cells or 293 cells expressing Myc-tagged wild type E-cadherin or its various extracellular domain deletion mutants were incubated with human erythrocyte GAPDH. The immunoprecipitates generated with anti-GAPDH antibody were analyzed by Western blotting with anti-Myc antibody. L, 1/100 of loaded cell membranes.
(PDF)

S24 Fig Effect of MEK inhibitors on Hs738 cells. Hs738 cells were cultured with inhibitors. After 2 days, the expressions of vimentin, SM-α-actin, and tubulin were analyzed by Western blot (upper). After 3 days, the cell growth was determined using MTT (lower). The values are means ± s.d. (n = 3).
(PDF)

S25 Fig Summary of tumor-stromal cell interactions in gastric cancer. (PDF)

S26 Fig Synthesis of MEK inhibitor I derivatives. To a solution of carboxylic acid 2 (ref 36) (10.5 mg, 29.4 mmol) was added DIPEA (5.6 mL, 32.2 mmol) and HATU (12.2 mg, 32.1 mmol) at 0°C and the resulting mixture was stirred for 30 min at room temperature. Then, MEK inhibitor I 1 (10.0 mg, 26.7 mmol) in DMF (0.7 mL) was added to the solution at 0°C, and the mixture was stirred for 36 h at room temperature and concentrated *in vacuo*. The residue was purified by preparative TLC (16.7% MeOH/CHCl_3_) to give the probe molecule 3 in 42% yield (7.8 mg, 10.9 mmol, ca. 84:16 mixture of geometric isomers of olefin) as a colorless amorphous solid. The control compound 5 (mixture with unidentified by-products, a pale yellow amorphous solid, 6.1 mg, < 12.6 mmol) was synthesized according to the procedure described for the preparation of 3 using carboxylic acid 4 (4.6 mL, 32.4 mmol), and purified by preparative TLC (9.1% MeOH/CHCl_3_). DIPEA, *N*,*N*-diisopropylethylamine; HATU: 1-[bis(dimethylamino)methylene]- 1*H*-1,2,3-triazolo[4,5-*b*]pyridinium 3-oxid hexafluorophosphate.
(PDF)
