# Supplementary figures and images for: Stromal Cells Positively and Negatively Modulate the Growth of Cancer Cells: Stimulation via the PGE2-TNFα-IL-6 Pathway and Inhibition via Secreted GAPDH-E-Cadherin Interaction
Source: PLoS One. 2015 Mar 18;10(3):e0119415. doi: 10.1371/journal.pone.0119415 (PMC4364666; doi:10.1371/journal.pone.0119415)

Figure S1

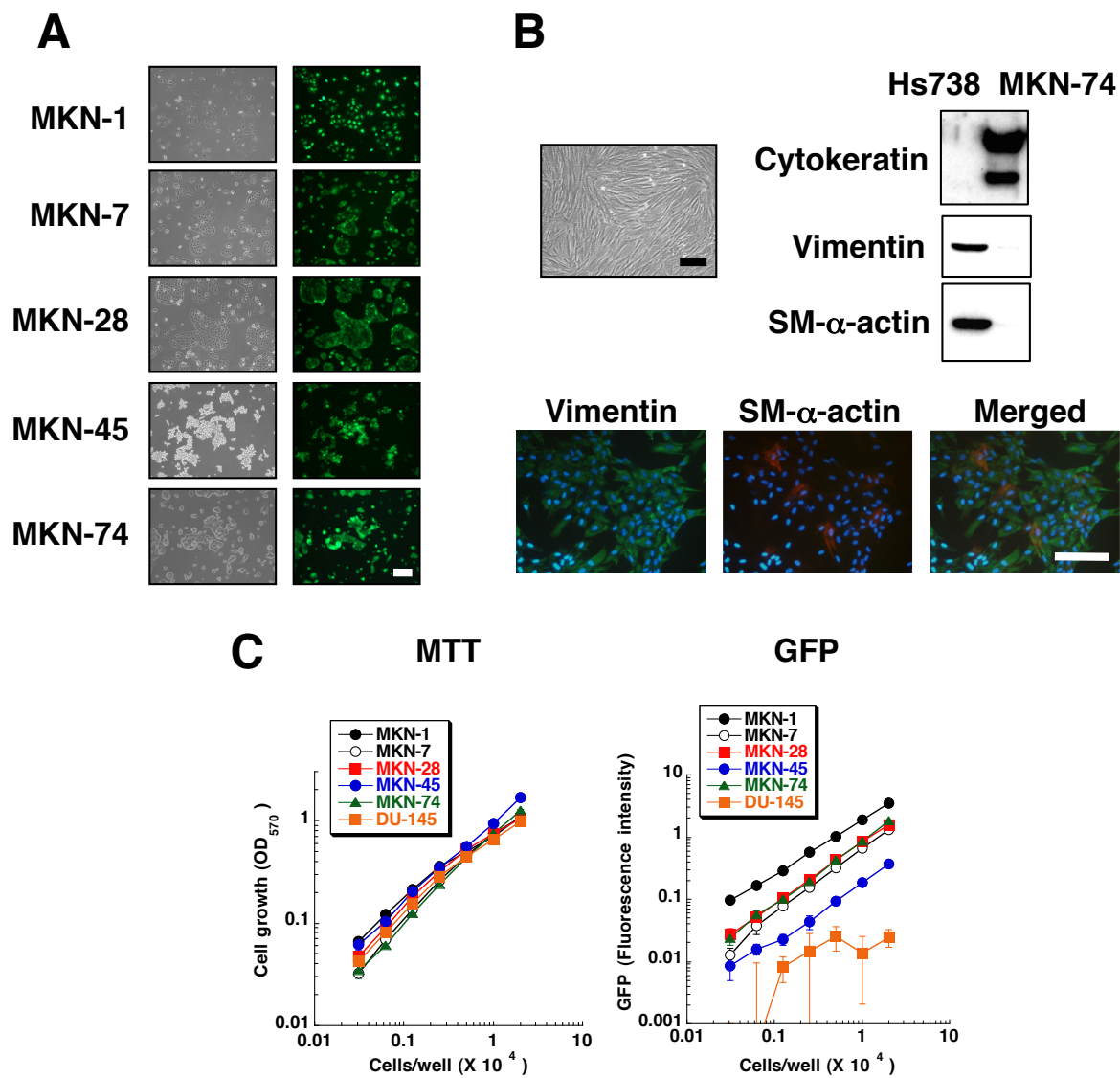

Supplement: S1 Fig — (A) Gastric cancer cell lines were stably transfected with a GFP expression vector. Photos were taken under phase contrast microscopy (left) and fluorescence microscopy (right). Scale bar is 200 μm. (B) Characteristics of Hs738 cells. Hs738 human gastric stromal cells were a mixture of fibroblasts expressing vimentin without SM-α-actin and myofibroblasts expressing both vimentin and SM-α-actin. A photo of Hs738 cells in upper left was taken under phase contrast microscopy. Western blots in upper right show that Hs738 cells express vimentin and SM-α-actin compared with MKN-74 gastric cancer cells expressing only cytokeratin. Lower panels show immunofluorescence staining of Hs738 cells with vimentin (green), SM-α-actin (red), and also DAPI staining (blue). Scale bar is 200 μm. (C) Cell growth of GFP-transfected cells. GFP-transfected gastric cancer cell lines and untransfected DU-145 prostate cancer cells were inoculated in 96-well plates with 10% FBS at the indicated numbers per well. After overnight incubation, the cell numbers were determined using MTT (left) or measuring GFP fluorescence intensity (right). Cell numbers correlated well with GFP fluorescence intensity as well as MTT in gastric cancer cell lines, but not in DU-145 cells without GFP transfection. The values are means ± s.d. (n = 3). (PDF) [file pone.0119415.s001.pdf]

Figure S2

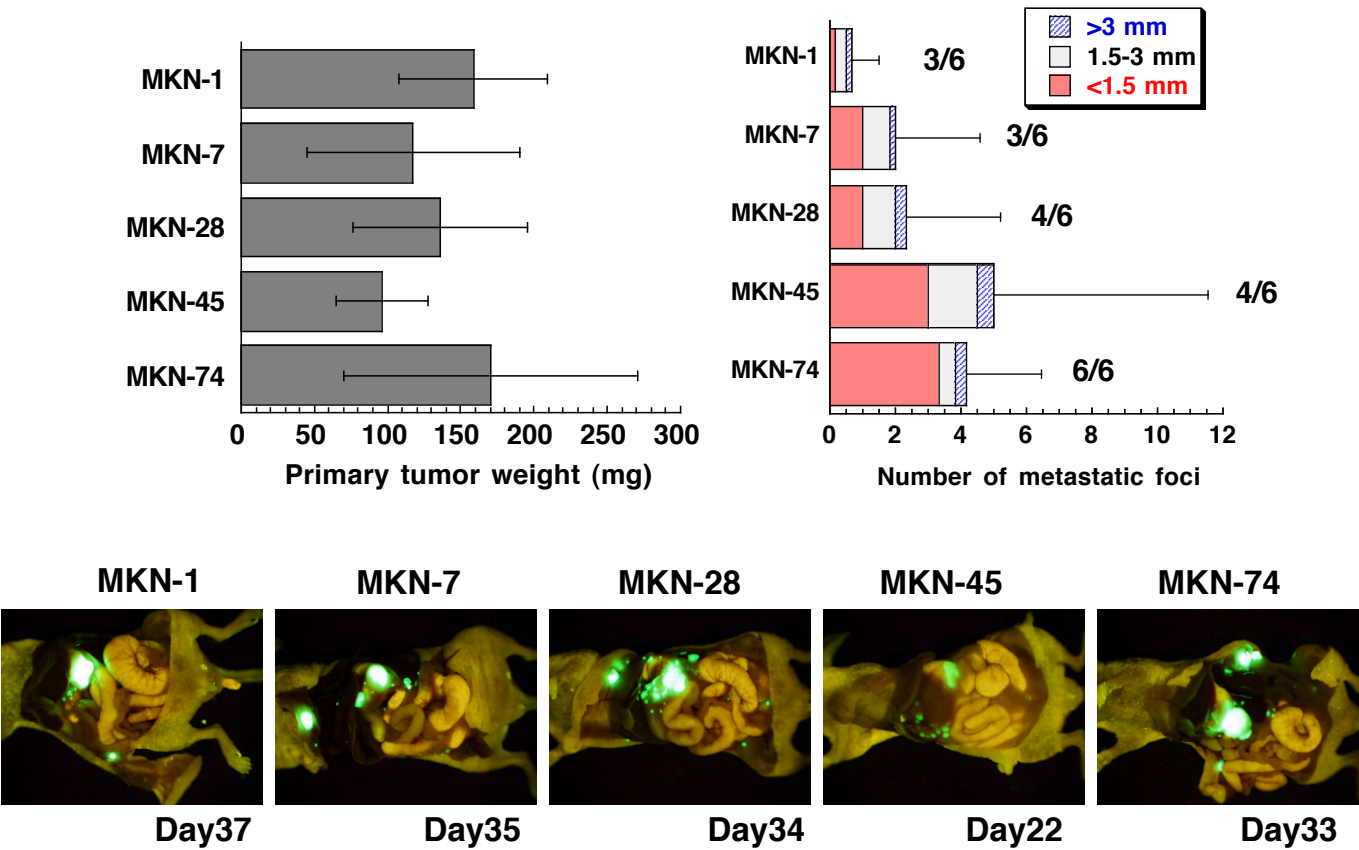

Supplement: S2 Fig — Gastric cancer cells were inoculated orthotopically into the stomach of female nude mice. The mice were sacrificed on days 37, 35, 34, 22, and 33 after the inoculation of MKN-1, 7, 28, 45, and 74 cells, respectively. The number of metastatic foci in the peritoneal cavity was counted under fluorescence microscopy (upper right) and the primary tumor was excised (upper left). Tumor size was classified as >3mm, 1.5–3 mm, and <1.5 mm. Numbers indicate animals positive for metastases. Representative photos of the peritoneal cavity are shown (lower panels). There are GFP-positive primary tumors and metastatic foci. The values are means ± s.d. (n = 6). (PDF) [file pone.0119415.s002.pdf]

Figure S3

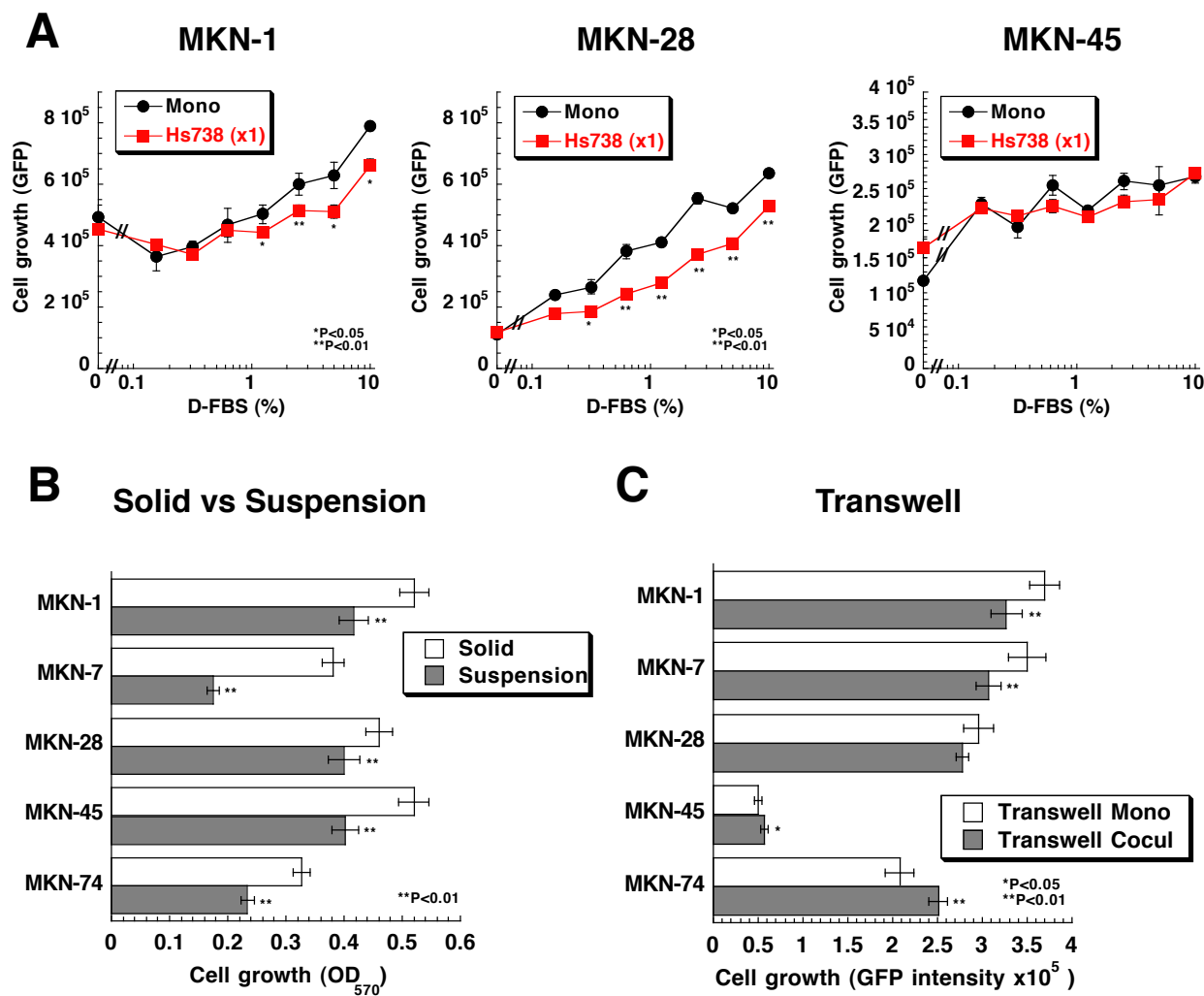

Supplement: S3 Fig — (A) Co-culture of gastric cancer cells and Hs738 cells. Gastric cancer cells were cultured alone (Mono) or co-cultured with Hs738 cells at a ratio (gastric cancer:Hs738) of 1:1 in the indicated concentrations of D-FBS. The growth of cancer cells was determined measuring GFP fluorescence intensity. The values are means ± s.d. (n = 3). (B) Gastric cancer cells were cultured in a suspension culture plate (Suspension) or a normal culture plate (Solid) for 3 days. The growth of cancer cells was determined using MTT. The values are means ± s.d. (n = 3). (C) In transwell plates, gastric cancer cells (inner wells) were cultured alone (Transwell Mono) or co-cultured with Hs738 cells (outer wells) (Transwell Cocul) for 3 days. The growth of cancer cells was determined measuring GFP fluorescence intensity. The values are means ± s.d. (n = 3). (PDF) [file pone.0119415.s003.pdf]

Figure S4

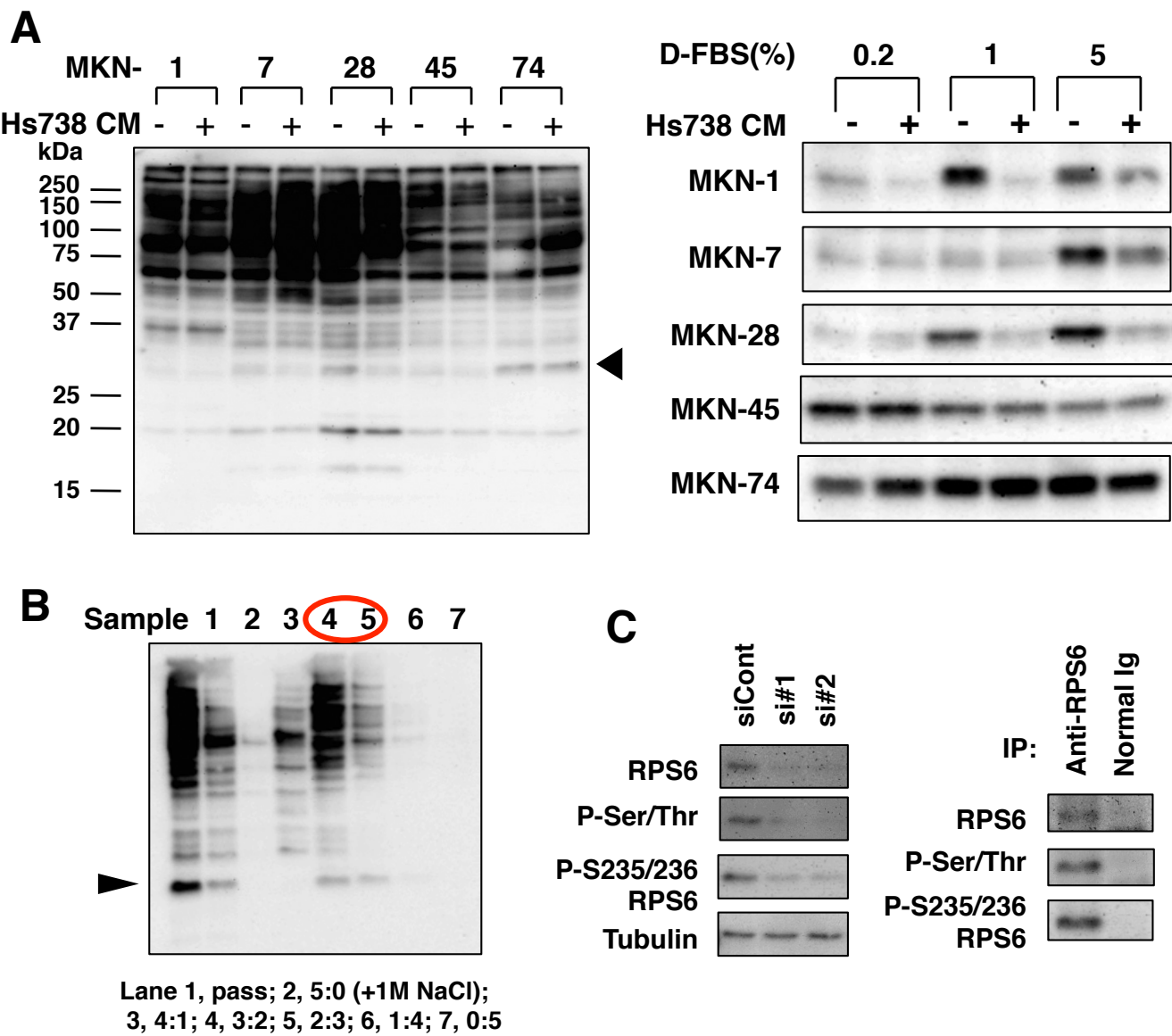

Supplement: S4 Fig — (A) Gastric cancer cells were cultured in the conditioned medium of Hs738 cells prepared by culturing Hs738 cells with 1% (left) or the indicated concentrations of D-FBS (right) (+) or control medium (-). Ser/Thr-phosphorylated proteins were detected by anti-phospho-Ser/Thr antibody (9624). An arrowhead indicates the position of the band of the right panel. (B) Partial purification of phosphorylated proteins in MKN-7 cells. Phosphorylated proteins were partially purified using an anionic column and detected by anti-phospho-Ser/Thr antibody (9624). The bands indicated by an arrowhead in lanes 4 and 5 were excised and analyzed by LC-MS/MS. The bands were deduced to be 14–3–3 protein epsilon and ribosomal protein S6 (RPS6). (C) Effect of siRNA against RPS6 on MNK-7 cells. MKN-7 cells were transfected with siRNAs against RPS6 (si#1 and si#2) for 2 days and the indicated proteins were analyzed by Western blot (left). MKN-7 cell lysates were immunoprecipitated with anti-RPS6 or normal Ig and the immunoprecipitates were detected by the indicated antibodies (right). (PDF) [file pone.0119415.s004.pdf]

Figure S6

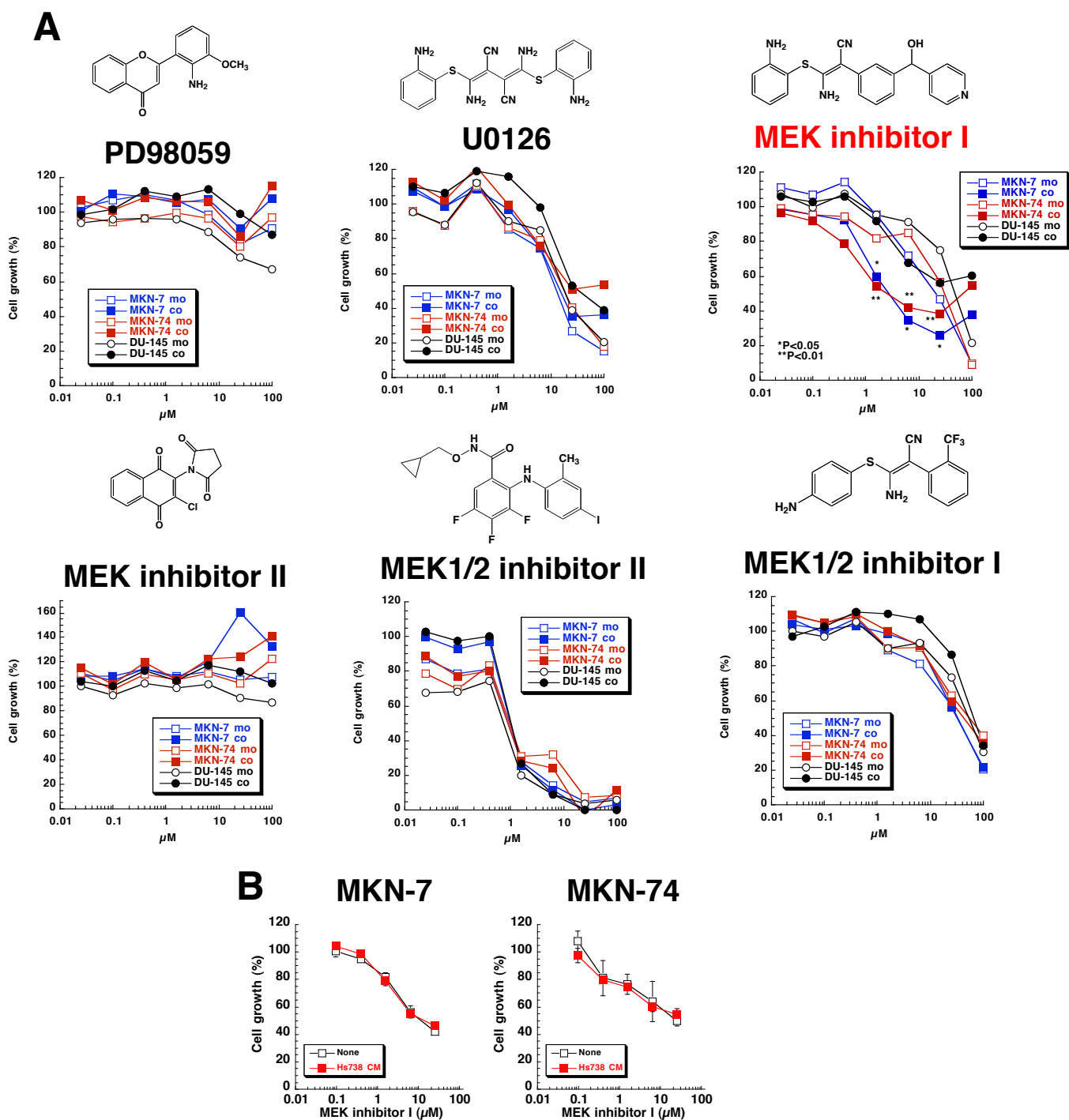

Supplement: S6 Fig — (A) GFP-expressing MKN-7, MKN-74, and DU-145 cells were cultured alone (mo) or co-cultured with Hs738 cells (co) for 3 days in the presence of inhibitors. The cell growth was determined measuring GFP fluorescence intensity. The values are means ± s.d. (n = 3). (B) MKN-7 and MKN-74 cells were cultured with MEK inhibitor I for 3 days in the presence or absence of Hs738 CM prepared by culturing Hs738 cells without inhibitors for 2 days. The cell growth was determined measuring GFP fluorescence intensity. The values are means ± s.d. (n = 3). Cell growth is expressed as a percentage of the value without test compounds in each culture condition. (PDF) [file pone.0119415.s006.pdf]

**Figure S7**

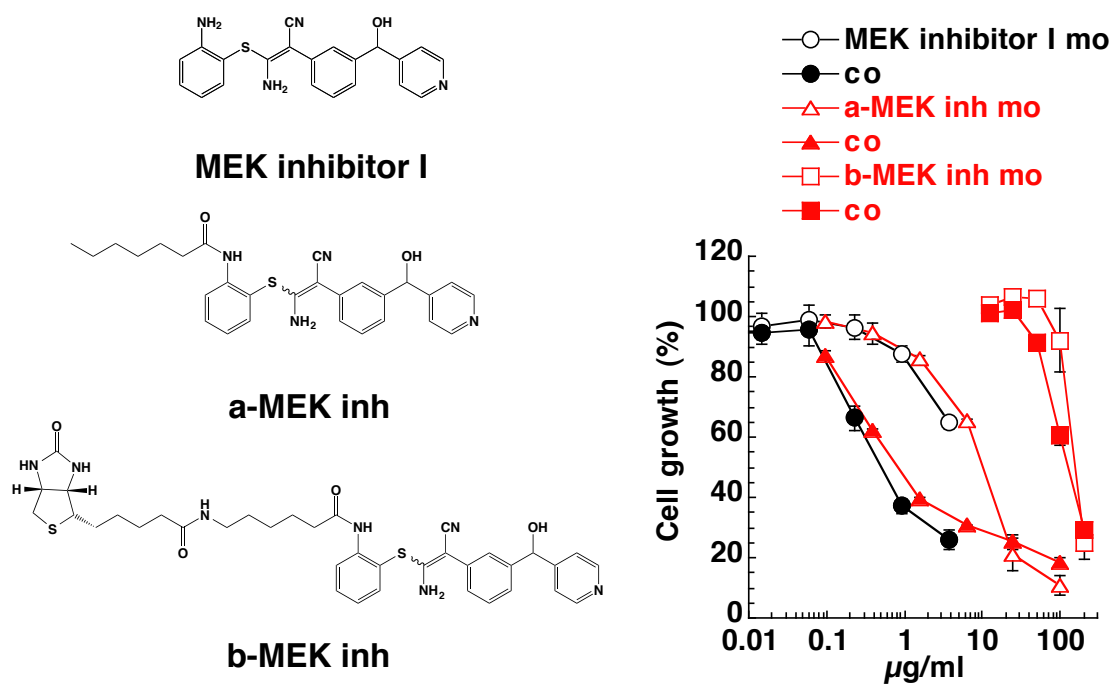

Supplement: S7 Fig — Acylated MEK inhibitor I (a-MEK inh) and biotinylated MEK inhibitor I (b-MEK inh) were synthesized. GFP-expressing MKN-7 cells were cultured alone (mo) or co-cultured with Hs738 cells (co) for 3 days in the presence of inhibitors. Cell growth was determined by measuring GFP fluorescence intensity. The values are means ± s.d. (n = 3). Cell growth is expressed as a percentage of the value without test compounds in each culture condition. Comparing the structures of MEK inhibitor I and U0126, amine of aniline moiety was modified. Because a-MEK inh sustained almost the same activity as MEK inhibitor I, we synthesized b-MEK inh by modifying the same site. (PDF) [file pone.0119415.s007.pdf]

**Figure S8**

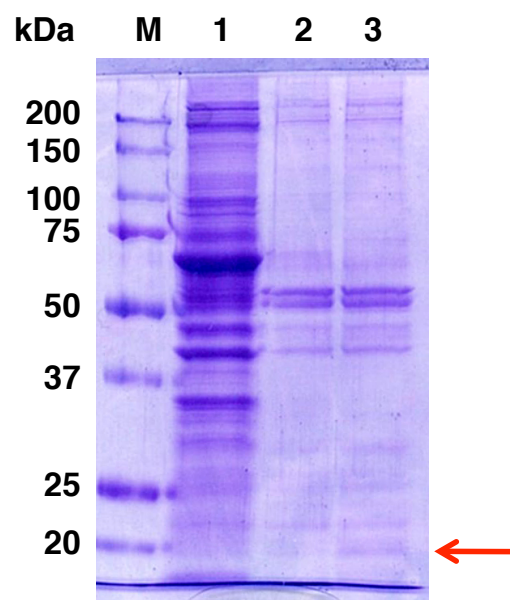

Supplement: S8 Fig — Hs738 cell extracts were incubated with b-MEK inh-pretreated Streptavidin resin and the bound proteins were analyzed by SDS-PAGE. Coomassie brilliant blue (CBB) staining revealed some bands specific to b-MEK inh (Arrow). M, marker; 1, Hs738 cell extracts; 2, negative control; 3, b-MEK inh-treated. Bands specific to b-MEK inh were analyzed by LC-MS/MS. LC-MS/MS analysis deduced several molecules such as RPL-18A and caveolin as candidates. (PDF) [file pone.0119415.s008.pdf]

Figure S9

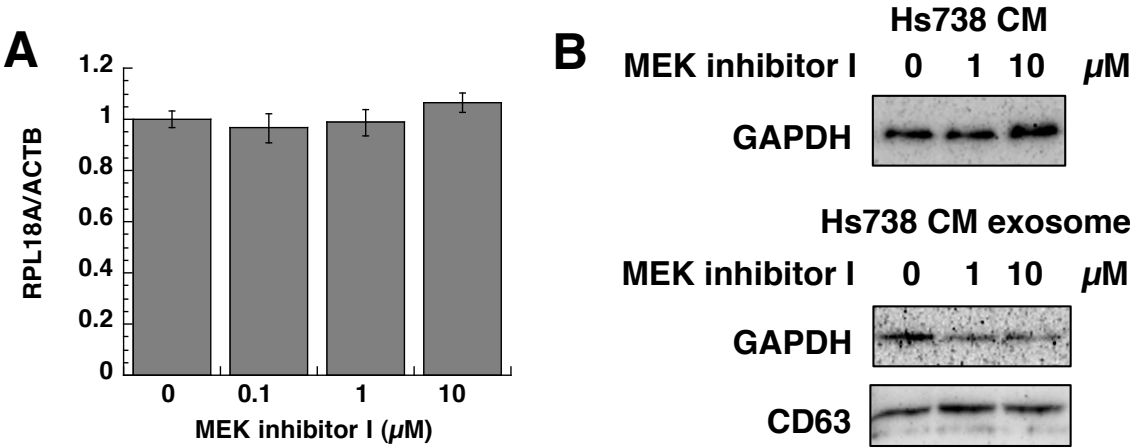

Supplement: S9 Fig — Hs738 cells were cultured with the indicated concentrations of MEK inhibitor I for 2 days. (A) Total RNAs were collected and RPL-18A mRNA levels were analyzed by real time RT-PCR using b-actin as a reference. (B) Exosomes were prepared from the cultured supernatant and analyzed by Western blot. CD63 is a marker of the exosome. (PDF) [file pone.0119415.s009.pdf]

Figure S10

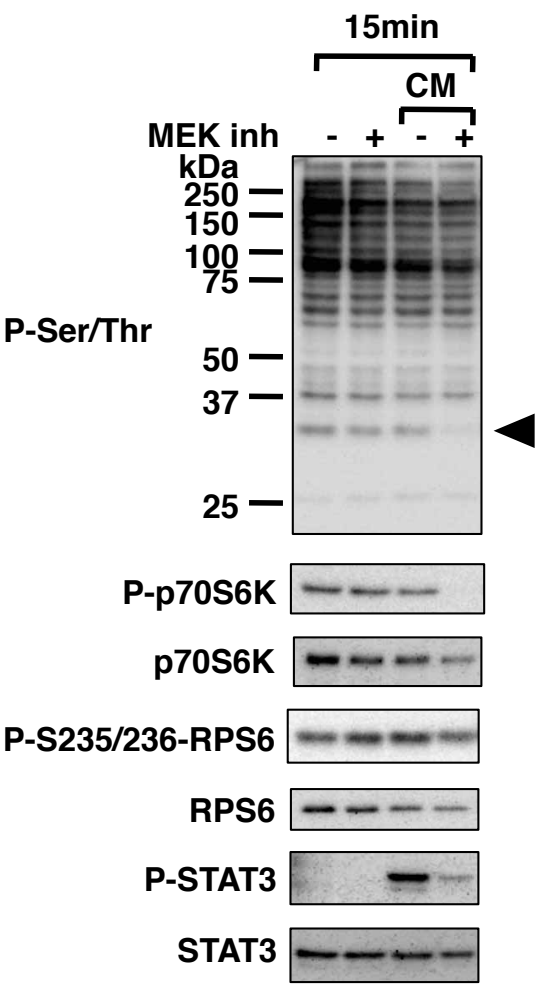

Supplement: S10 Fig — MKN-7 cells were cultured for the indicated times with or without 10 μM MEK inhibitor I or Hs738 CM prepared by culturing Hs738 cells with or without MEK inhibitor I for 2 days. The cell lysates were analyzed by Western blot with anti-phospho-(Ser/Thr) antibody (9624) and other indicated antibodies. An arrowhead indicates the position of RPS6 protein. (PDF) [file pone.0119415.s010.pdf]

Figure S12

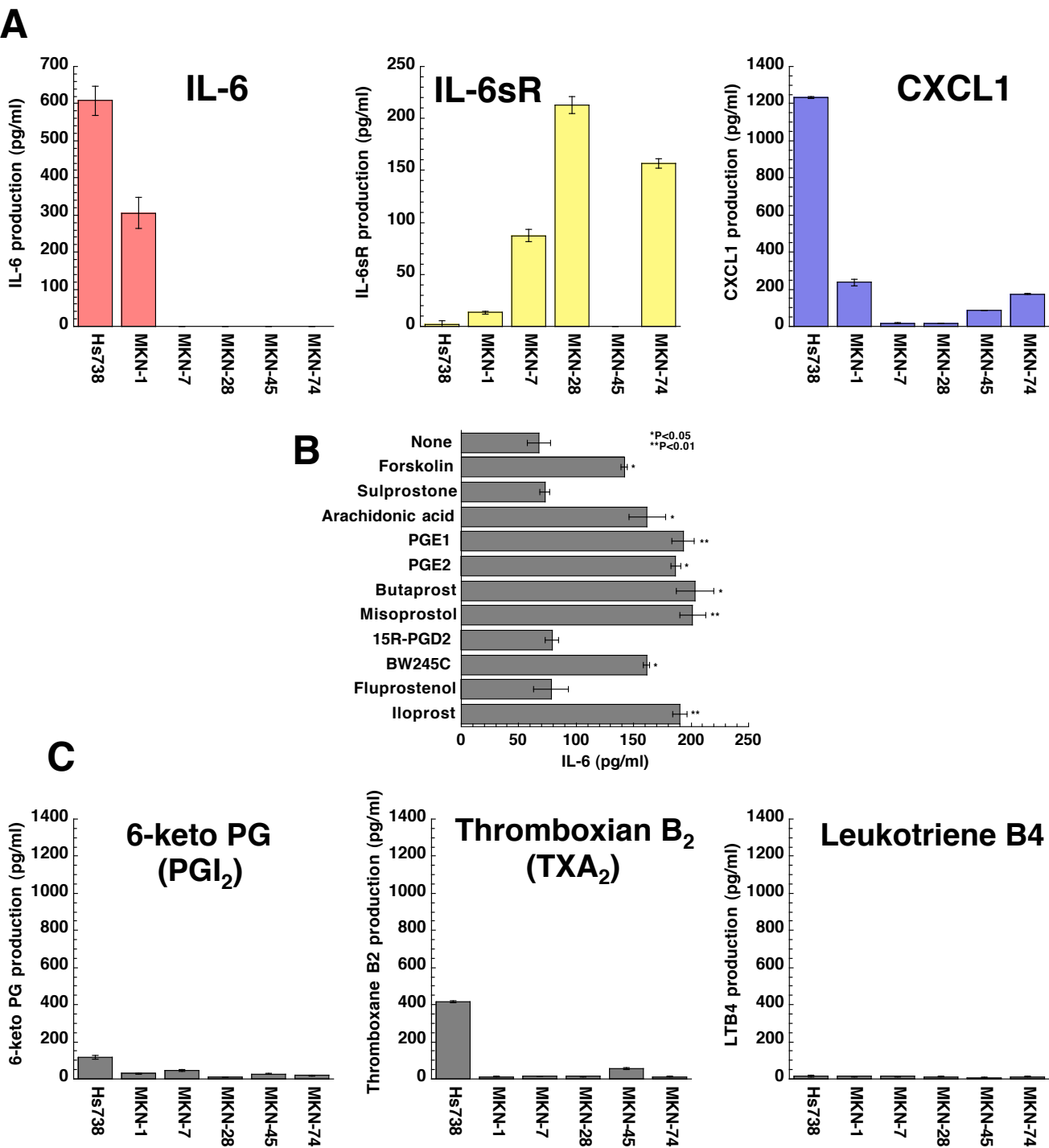

Supplement: S12 Fig — (A) IL-6, IL-6sR, and CXCL1 in CM. Cells were cultured for 2 days and the amounts of IL-6, IL-6sR, and CXCL1 in the CM were determined. The values are means ± s.d. (n = 3). (B) Effect of various prostanoids on IL-6 secretion. Hs738 cells were cultured with 10μM various prostanoids for 1 day. The amounts of IL-6 in the cultured supernatant were determined. The values are means ± s.d. (n = 3). (C) Prostanoids production in cells. The amounts of PGI2, TXA2, and leukotriene B4 in CM prepared from the indicated cells cultured for 2 days were determined. The values are means ± s.d. (n = 3). (PDF) [file pone.0119415.s012.pdf]

Figure S13

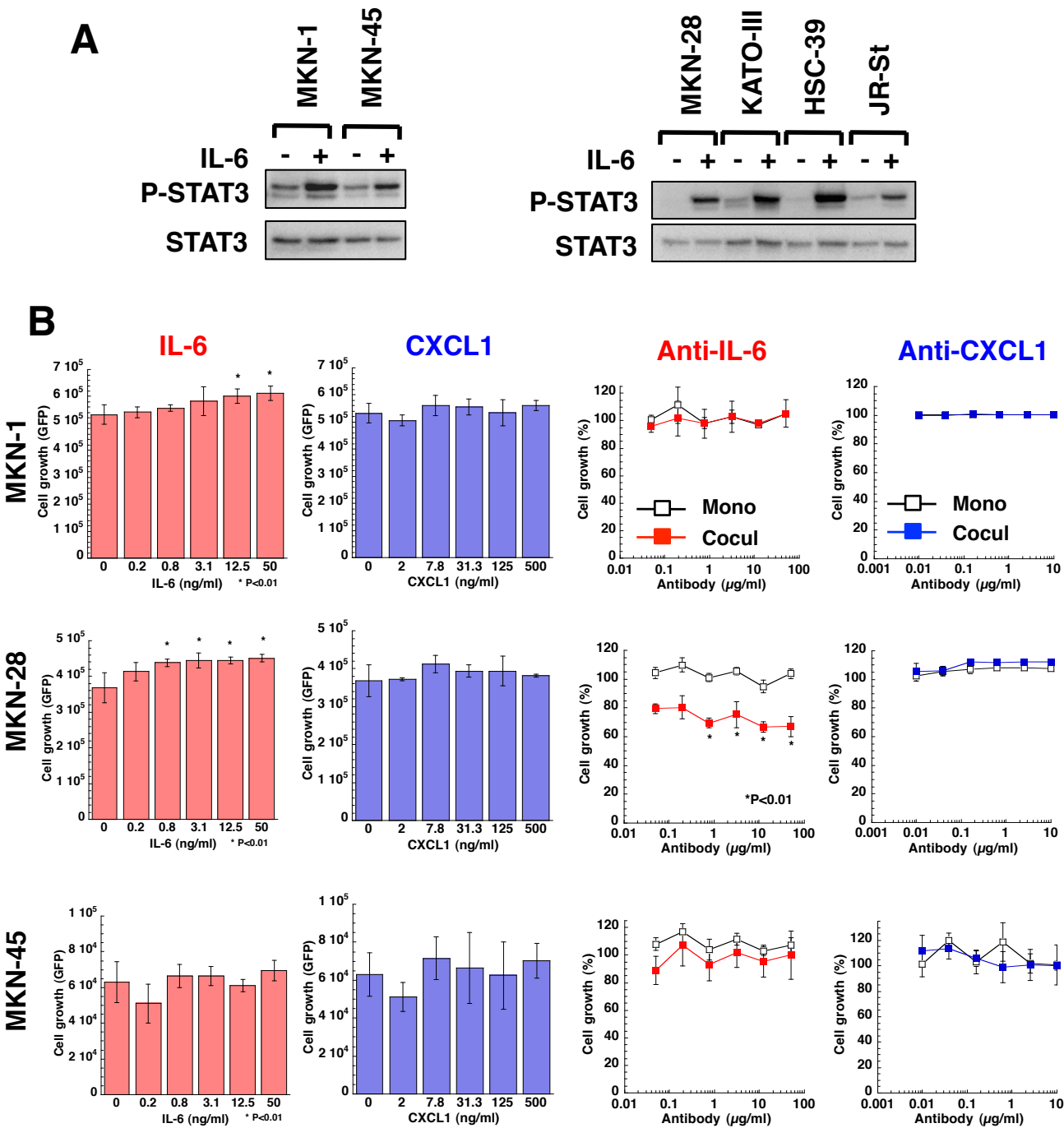

Supplement: S13 Fig — (A) STAT-3 activation in gastric cancer cells. Cells were cultured for 15 min with 50 ng/ml of IL-6. The activation of STAT3 was analyzed by Western blot. (B) Effect of IL-6, CXCL1, anti-IL-6, and anti-CXCL-1 antibodies on growth of gastric cancer cells. Cells were cultured with IL-6, CXCL1, anti-IL-6, or anti-CXCL-1 antibodies (Mono) for 3 days or co-cultured with Hs738 cells (Cocul) for 3 days. Cell growth was determined measuring GFP fluorescence intensity. The values are means ± s.d. (n = 3). For anti-IL-6 and anti-CXCL-1 antibodies, cell growth is expressed as a percentage of the value without antibodies in each culture condition. (PDF) [file pone.0119415.s013.pdf]

Figure S14

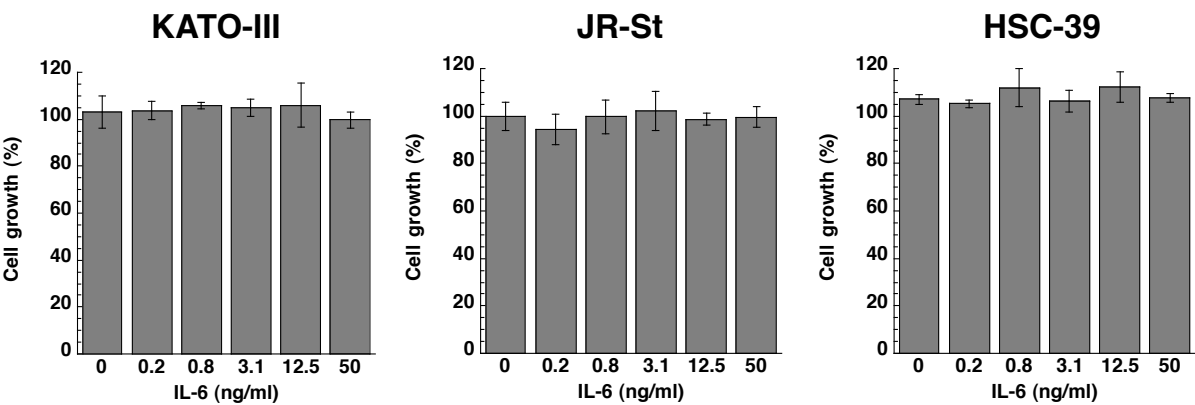

Supplement: S14 Fig — Cells were cultured with IL-6 for 3 days. Cell growth was determined using MTT. The values are means ± s.d. (n = 3). (PDF) [file pone.0119415.s014.pdf]

**Figure S15**

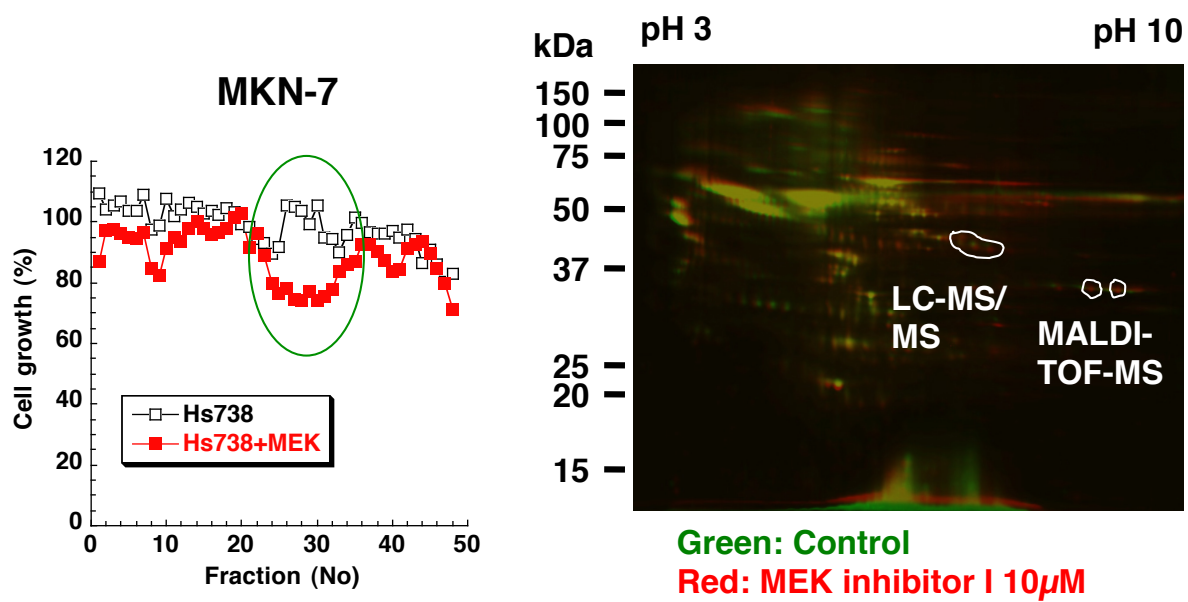

Supplement: S15 Fig — Hs738 CM prepared by culturing Hs738 cells with or without 10 μM MEK inhibitor I for 3 days was concentrated and separated by gel filtration. MKN-7 cells were cultured with the fractionated CM concentrate for 3 days and the cell growth was determined using MTT (left). The fractions with growth inhibitory activity (green circle) were pooled and applied onto 2D gel electrophoresis (right). Spots, in which expressions in MEK inhibitor I-treated cells were higher than control, were analyzed by LC-MS/MS or MALDI-TOF-MS. LC-MS/MS analysis deduced PAI-precursor protein and α-enolase and MALDI-TOF-MS deduced GAPDH. (PDF) [file pone.0119415.s015.pdf]

Figure S16

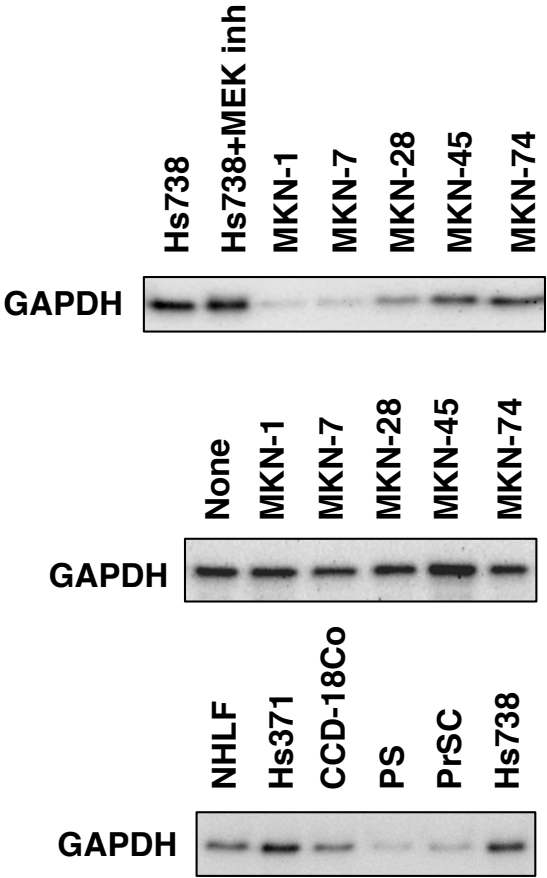

Supplement: S16 Fig — Cells were cultured with or without 10 μM MEK inhibitor I for 2 days (upper). Hs738 cells were cultured for 1 day in CM prepared by culturing gastric cancer cells for 2 days (middle). Various stromal cells were cultured for 2 days (lower). GAPDH in the cultured supernatant was analyzed by Western blot. (PDF) [file pone.0119415.s016.pdf]

Figure S17

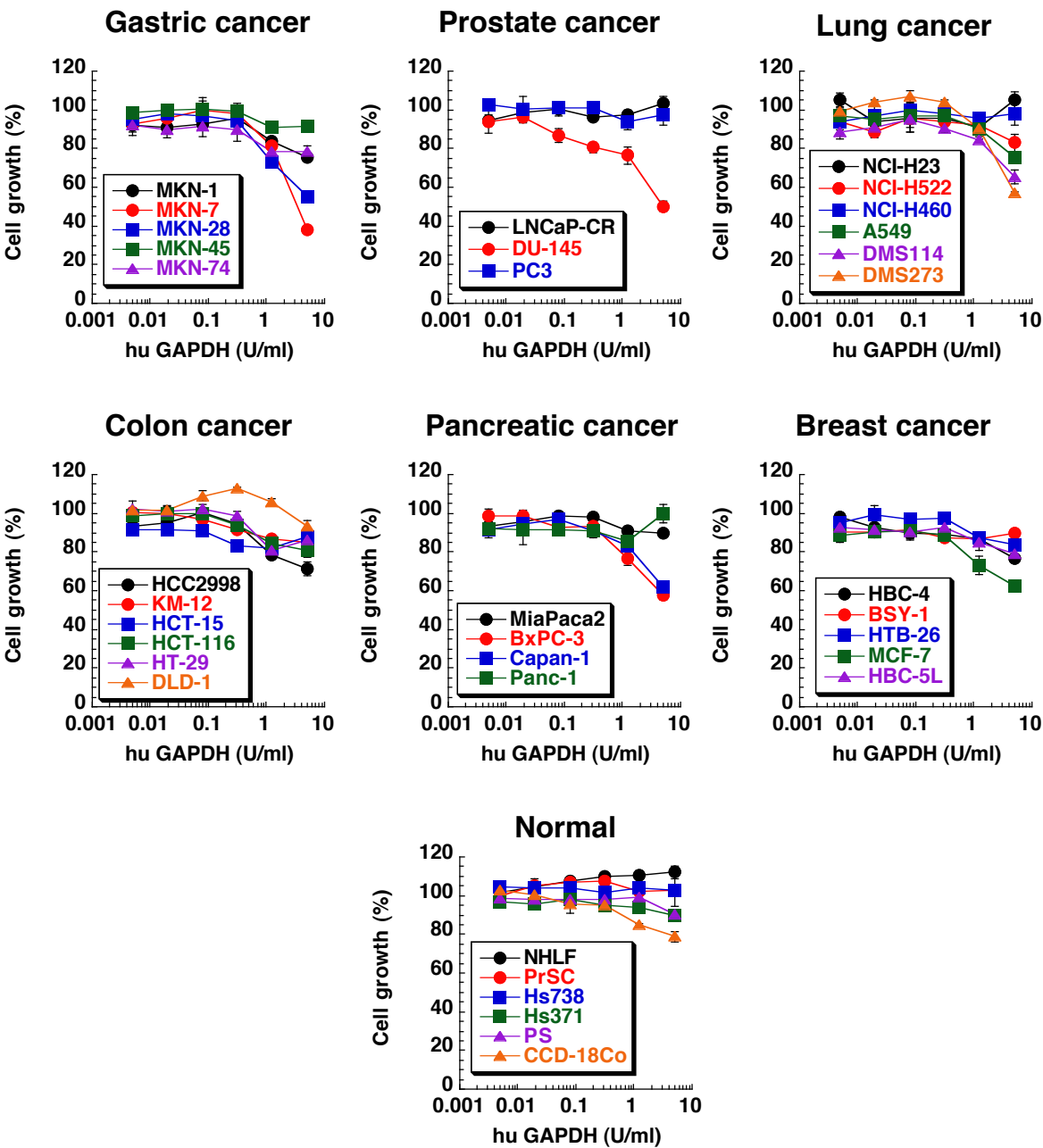

Supplement: S17 Fig — Cells were cultured with human erythrocyte GAPDH for 3 days. Cell growth was determined using MTT. The values are means ± s.d. (n = 3). Cell growth is expressed as a percentage of the value without hu GAPDH. (PDF) [file pone.0119415.s017.pdf]

Figure S18

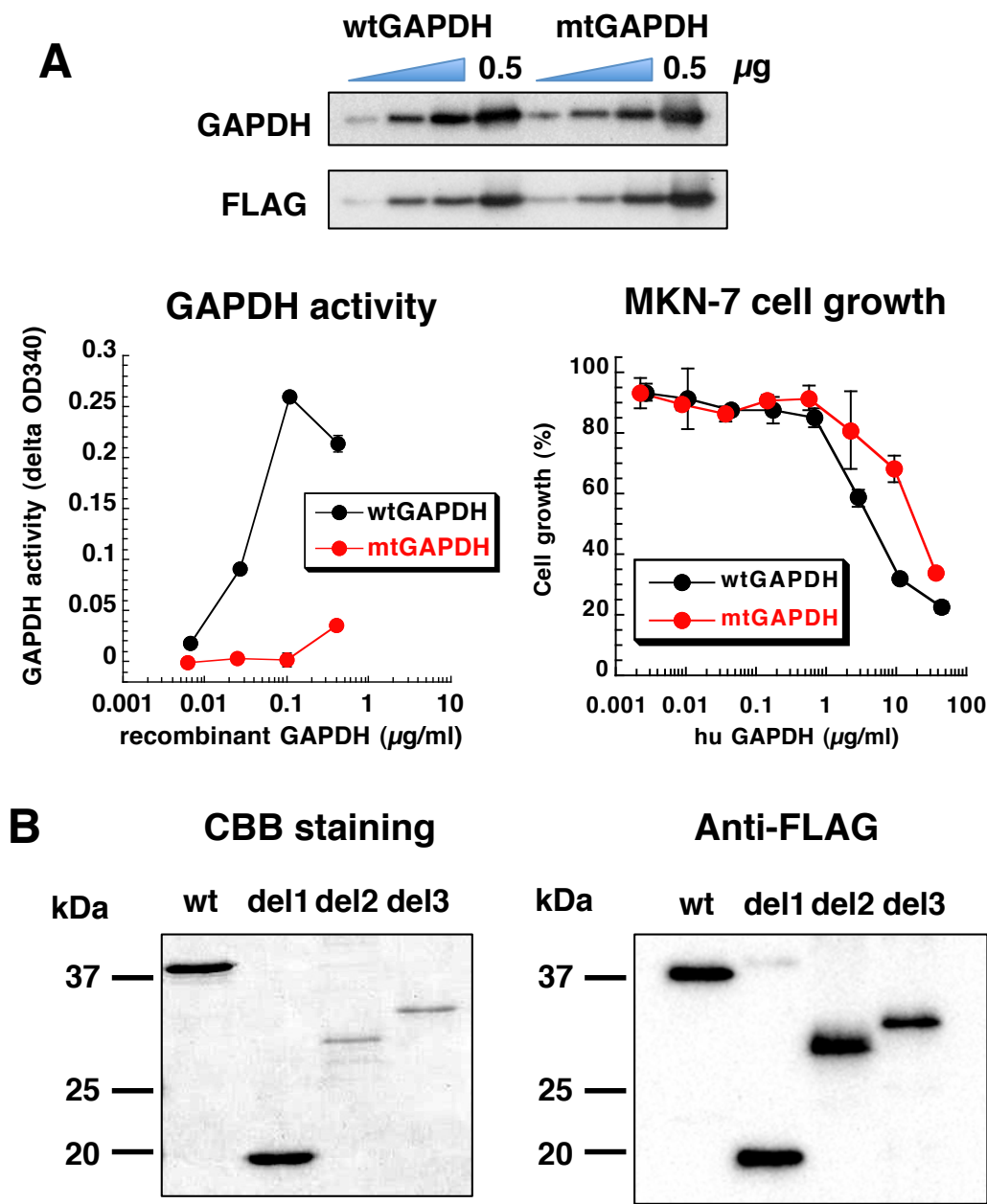

Supplement: S18 Fig — (A) Human recombinant wild type (wt) and mutant (mt) GAPDH were detected by Western blot using anti-GAPDH and anti-FLAG antibodies (upper). GAPDH enzyme activity of the recombinant GAPDH was measured. MKN-7 cells were cultured with wt and mt GAPDH for 3 days. Cell growth was determined using MTT. The values are means ± s.d. (n = 3). Cell growth is expressed as a percentage of the value without hu GAPDH. (B) Human recombinant wild type (wt) and deletion mutants of GAPDH were constructed using ClearColiBL21. GAPDH del1 lacks the C-terminal domain, GAPDH del2 lacks 4–81 amino acids in the N-terminal domain, and GAPDH del3 lacks 98–152 amino acids in the N-terminal domain. Estimated sizes are 38, 19.3, 27.1, and 30.3 kDa for wt, del1, del2, and del3, respectively. They were separated by SDS-PAGE and assessed by CBB staining (left) and Western blot with anti-FLAG antibody (right). (PDF) [file pone.0119415.s018.pdf]

Figure S20

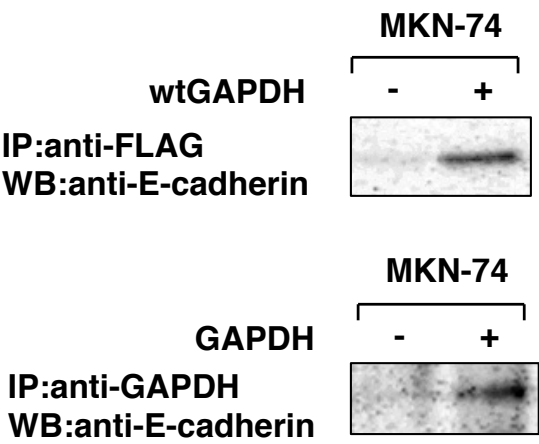

Supplement: S20 Fig — Cell membranes of MKN-74 cells were incubated with FLAG-tagged human recombinant wild type GAPDH or human erythrocyte GAPDH. The immunoprecipitates generated by the indicated antibodies were analyzed by Western blot with anti-E-cadherin antibody. No band was detected by anti-integrin β1 antibody. (PDF) [file pone.0119415.s020.pdf]

Figure S21

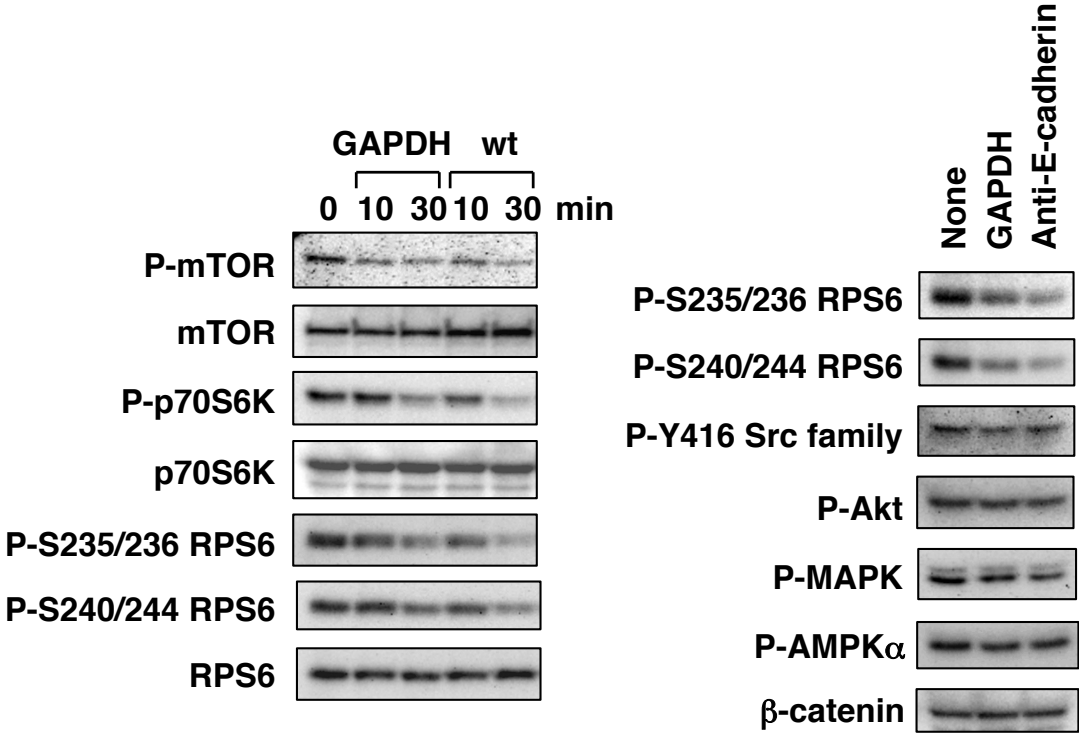

Supplement: S21 Fig — MKN-7 cells were cultured with human erythrocyte GAPDH at 5 U/ml or recombinant wild type (wt) GAPDH at 5 μg/ml for the indicated times. mTOR-p70S6K pathway was analyzed by Western blot (left). MKN-7 cells were cultured with human erythrocyte GAPDH at 5 U/ml or anti-E-cadherin antibody at 1 μg/ml for 30 min. Activated forms of the indicated proteins and b-catenin were analyzed by Western blot (right). (PDF) [file pone.0119415.s021.pdf]

Figure S23

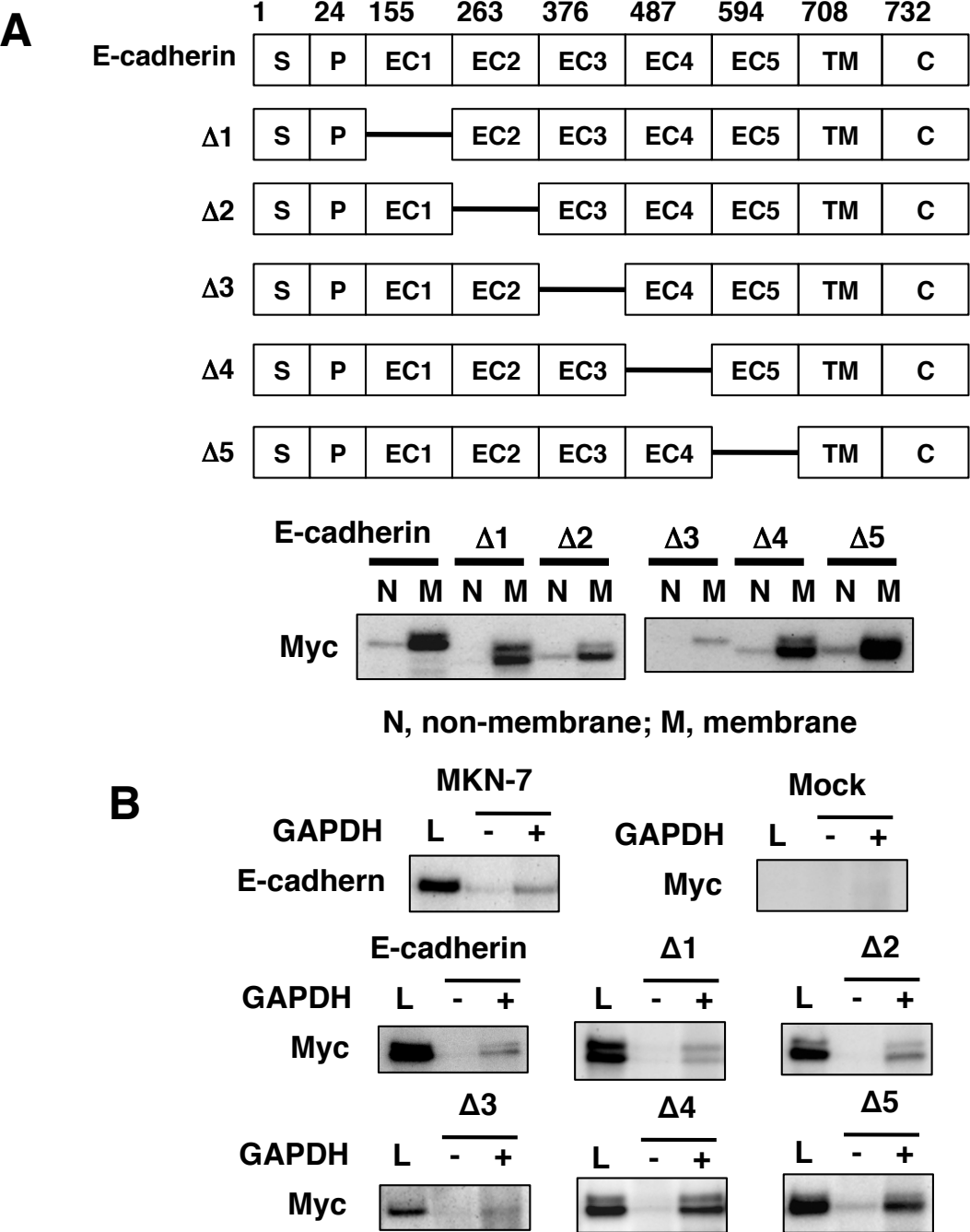

Supplement: S23 Fig — (A) 293 cells expressing Myc-tagged wild-type E-cadherin or its various extracellular domain deletion mutants as illustrated above were established. Non-membrane (N) and membrane (M) extracts were analyzed by Western blot. All cells expressed E-cadherin or its mutants on membranes. E-cadherin has five extracellular repeats (EC1-EC5). The numbers above the columns indicate the amino acid number, counting from the start of the coding region. S. signal peptide; P, propeptide; TM, transmembrane domain; C, cytoplasmic domain. The illustration was modified from ref 35. (B) Cell membranes of MKN-7 cells or 293 cells expressing Myc-tagged wild type E-cadherin or its various extracellular domain deletion mutants were incubated with human erythrocyte GAPDH. The immunoprecipitates generated with anti-GAPDH antibody were analyzed by Western blotting with anti-Myc antibody. L, 1/100 of loaded cell membranes. (PDF) [file pone.0119415.s023.pdf]

Figure S24

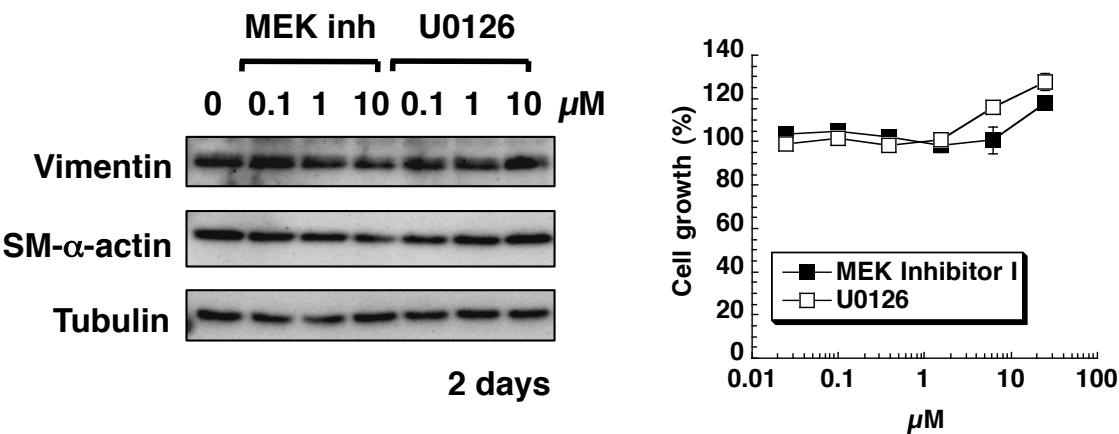

Supplement: S24 Fig — Hs738 cells were cultured with inhibitors. After 2 days, the expressions of vimentin, SM-α-actin, and tubulin were analyzed by Western blot (upper). After 3 days, the cell growth was determined using MTT (lower). The values are means ± s.d. (n = 3). (PDF) [file pone.0119415.s024.pdf]

Figure S25

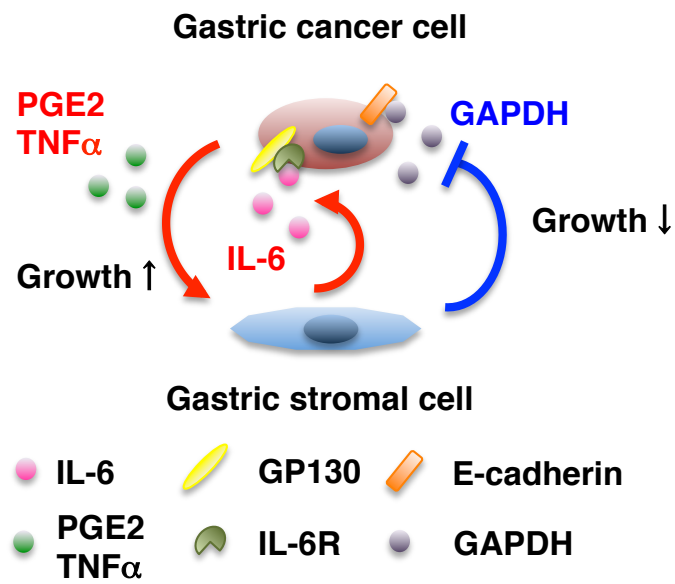

Supplement: S25 Fig — (PDF) [file pone.0119415.s025.pdf]

Figure S26

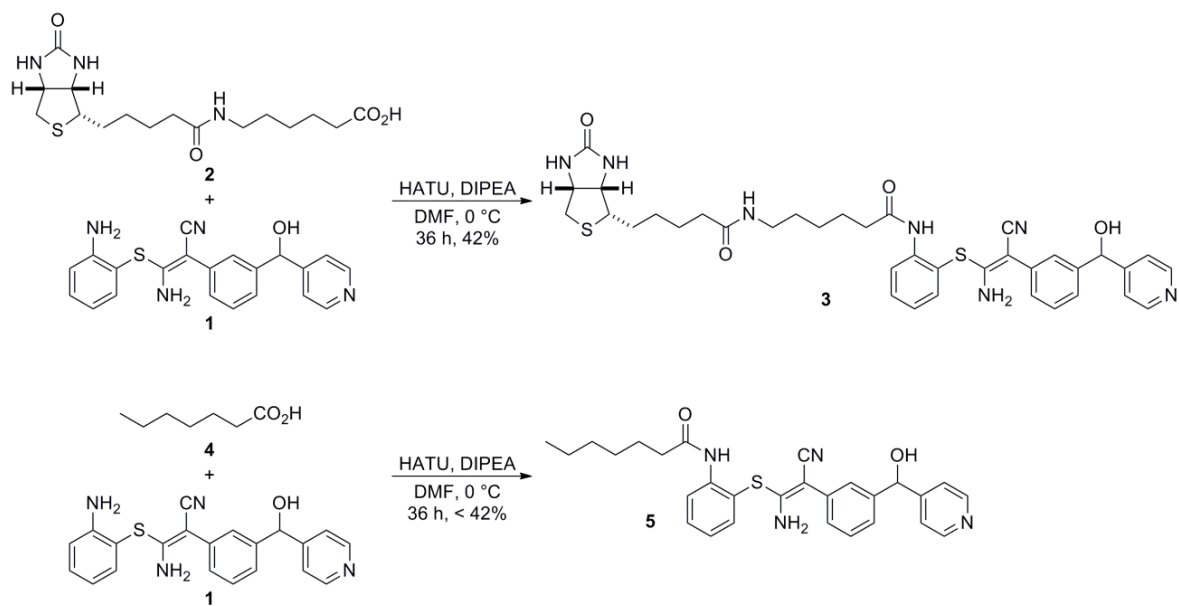

Supplement: S26 Fig — To a solution of carboxylic acid 2 (ref 36) (10.5 mg, 29.4 mmol) was added DIPEA (5.6 mL, 32.2 mmol) and HATU (12.2 mg, 32.1 mmol) at 0°C and the resulting mixture was stirred for 30 min at room temperature. Then, MEK inhibitor I 1 (10.0 mg, 26.7 mmol) in DMF (0.7 mL) was added to the solution at 0°C, and the mixture was stirred for 36 h at room temperature and concentrated in vacuo. The residue was purified by preparative TLC (16.7% MeOH/CHCl3) to give the probe molecule 3 in 42% yield (7.8 mg, 10.9 mmol, ca. 84:16 mixture of geometric isomers of olefin) as a colorless amorphous solid. The control compound 5 (mixture with unidentified by-products, a pale yellow amorphous solid, 6.1 mg, < 12.6 mmol) was synthesized according to the procedure described for the preparation of 3 using carboxylic acid 4 (4.6 mL, 32.4 mmol), and purified by preparative TLC (9.1% MeOH/CHCl3). DIPEA, N,N-diisopropylethylamine; HATU: 1-[bis(dimethylamino)methylene]- 1H-1,2,3-triazolo[4,5-b]pyridinium 3-oxid hexafluorophosphate. (PDF) [file pone.0119415.s026.pdf]
