# Supplementary material for: Stromal Cells Positively and Negatively Modulate the Growth of Cancer Cells: Stimulation via the PGE2-TNFα-IL-6 Pathway and Inhibition via Secreted GAPDH-E-Cadherin Interaction
Source: PLoS One. 2015 Mar 18;10(3):e0119415. doi: 10.1371/journal.pone.0119415 (PMC4364666; doi:10.1371/journal.pone.0119415)
Supplement: S5 Fig — MKN-7 and MKN-74 cells were cultured with or without Hs738 cells for 3 days in the presence of various inhibitors (about 300 compound; for details, http://scads.jfcr.or.jp/kit/kit.html) at 1 and 10 μM. The growth of cancer cells was expressed as a heat map. An arrow indicates the position of MEK inhibitor I. (PDF) [file pone.0119415.s005.pdf]

Figure S5

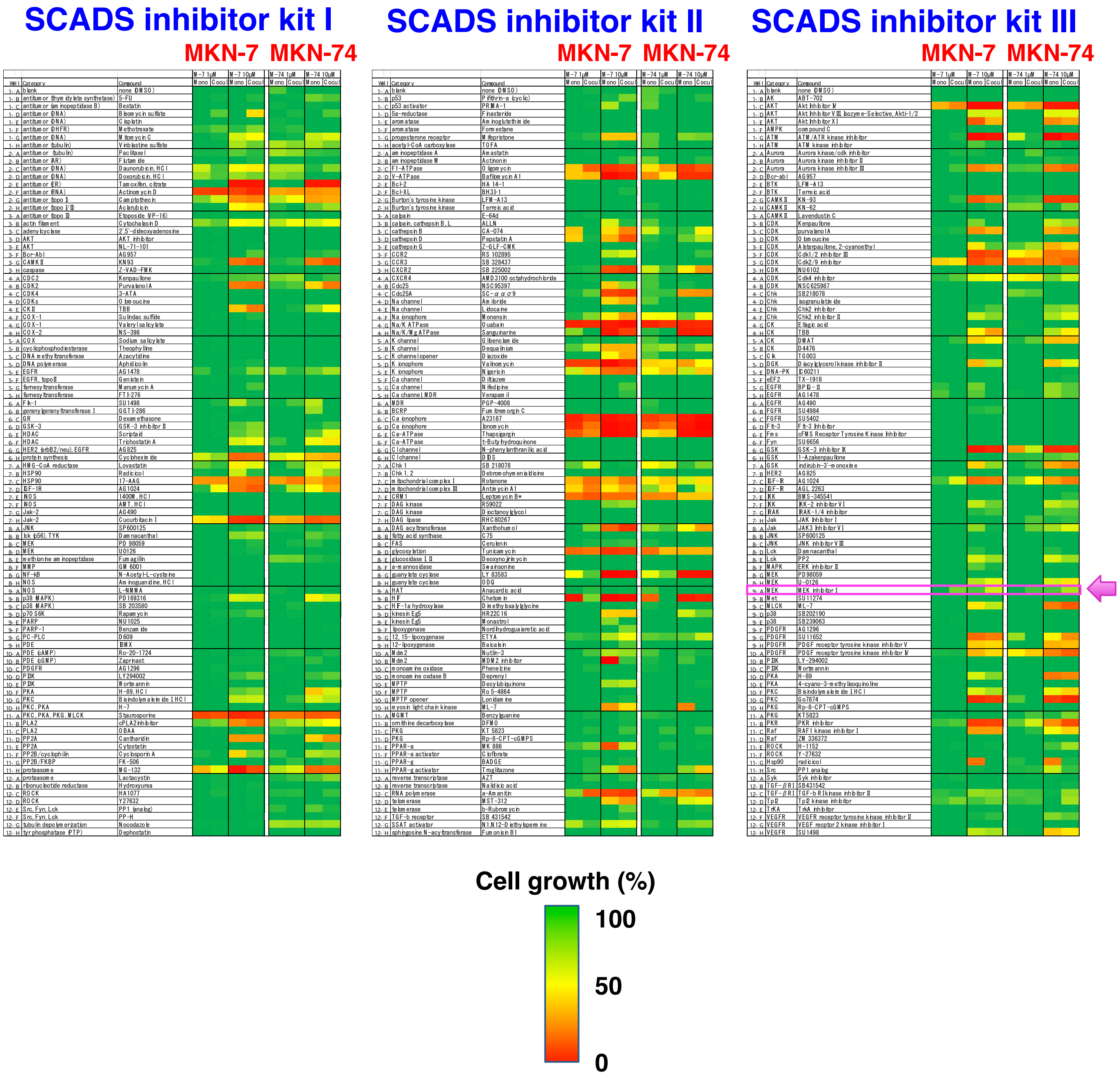
SCADS inhibitor kit I
MKN-7
MKN-74
SCADS inhibitor kit II
MKN-7
MKN-74
SCADS inhibitor kit III
MKN-7
MKN-74
Cell growth (%)
100
50
0
