# Supplementary material for: Stromal Cells Positively and Negatively Modulate the Growth of Cancer Cells: Stimulation via the PGE2-TNFα-IL-6 Pathway and Inhibition via Secreted GAPDH-E-Cadherin Interaction
Source: PLoS One. 2015 Mar 18;10(3):e0119415. doi: 10.1371/journal.pone.0119415 (PMC4364666; doi:10.1371/journal.pone.0119415)
Supplement: S11 Fig — Hs738 cells were cultured with or without 10 μM MEK inhibitor I for 2 days. The cultured supernatants were applied onto human cytokine antibody array (C series 2000; for details, http://www.raybiotech.com). Arrows indicate the positions of IL-6 and CXCL1 duplicated spots. Lower panels are lists of the arrays. (PDF) [file pone.0119415.s011.pdf]

Figure S11

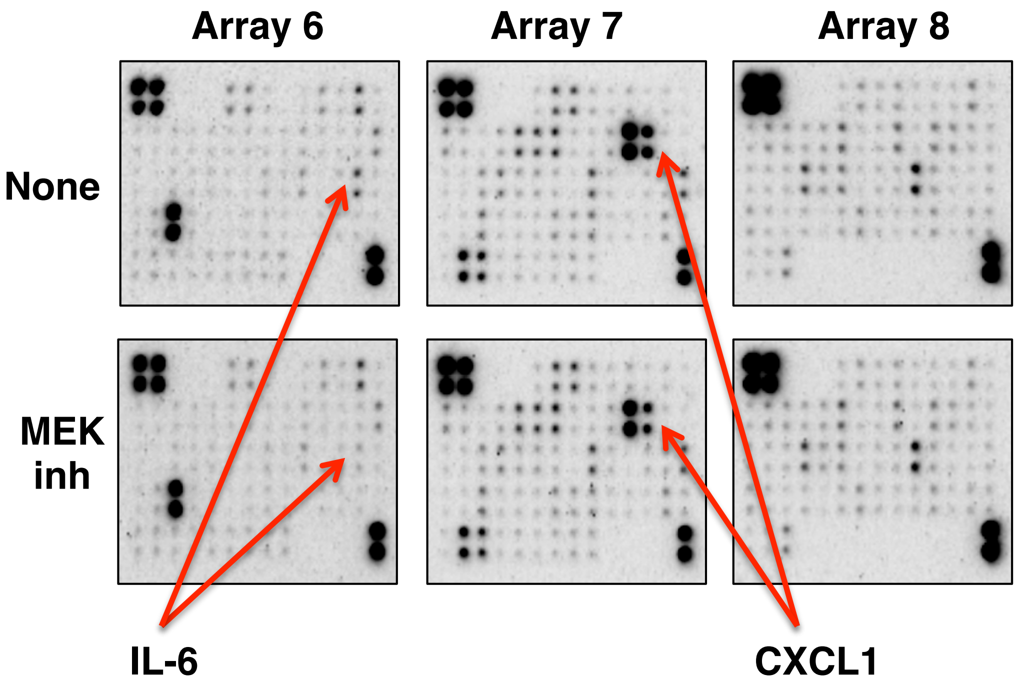

RayBio Human Cytokine Antibody Array 6 (60)

	a	b	c	d	e	f	g	h	i	j	k	l	m	n
1	POS	POS	NEG	NEG	Blank	Angiogenin	BDNF	BLC	BMP-4	BMP-6	CK β 8-1	CNTF	EGF	Eotaxin
2	POS	POS	NEG	NEG	Blank	Angiogenin	BDNF	BLC	BMP-4	BMP-6	CK β 8-1	CNTF	EGF	Eotaxin
3	Eotaxin-2	Eotaxin-3	FGF-6	FGF-7	Fit-3 Ligand	Fractalkine	GCP-2	GDNF	GM-CSF	I-309	IFN-γ	IGFBP-1	IGFBP-2	IGFBP-4
4	Eotaxin-2	Eotaxin-3	FGF-6	FGF-7	Fit-3 Ligand	Fractalkine	GCP-2	GDNF	GM-CSF	I-309	IFN-γ	IGFBP-1	IGFBP-2	IGFBP-4
5	IGF-I	IL-10	IL-13	IL-15	IL-16	IL-1 α	IL-1 β	IL-1ra	IL-2	IL-3	IL-4	IL-5	IL-6	IL-7
6	IGF-I	IL-10	IL-13	IL-15	IL-16	IL-1 α	IL-1 β	IL-1ra	IL-2	IL-3	IL-4	IL-5	IL-6	IL-7
7	Leptin	LIGHT	MCP-1	MCP-2	MCP-3	MCP-4	M-CSF	MDC	MIG	MIP-1 δ	MIP-3 α	NAP-2	NT-3	PARC
8	Leptin	LIGHT	MCP-1	MCP-2	MCP-3	MCP-4	M-CSF	MDC	MIG	MIP-1 δ	MIP-3 α	NAP-2	NT-3	PARC
9	PDGF-BB	RANTES	SCF	SDF-1	TARC	TFG−β 1	TFG-β 3	TNF-α	TNF-β	Blank	Blank	Blank	Blank	POS
10	PDGF-BB	RANTES	SCF	SDF-1	TARC	TFG−β 1	TFG-β 3	TNF-α	TNF-β	Blank	Blank	Blank	Blank	POS

RayBio Human Cytokine Antibody Array 7 (60)

	a	b	c	d	e	f	g	h	i	j	k	l	m	n
1	POS	POS	NEG	NEG	Blank	Acrp30AgRP		Angiopoietin-2	Amphiregulin	Axl	bFGF	b-NGF	BTC	CCL-28
2	POS	POS	NEG	NEG	Blank	Acrp30AgRP		Angiopoietin-2	Amphiregulin	Axl	bFGF	b-NGF	BTC	CCL-28
3	CTACK	Dtk	EGF-R	ENA-78	Fas/TNFRSF6	FGF-4	FGF-9	GCSF	GITR-Ligand	GITR	GRO	GRO-α	HCC-4	HGF
4	CTACK	Dtk	EGF-R	ENA-78	Fas/TNFRSF6	FGF-4	FGF-9	GCSF	GITR-Ligand	GITR	GRO	GRO-α	HCC-4	HGF
5	ICAM-1	ICAM-3	IGFBP-3	IGFBP-6	IGF-I SR	IL-1 R4/ST2	IL-1 RI	IL-11	IL-12p40	IL-12p70	IL-17	IL-2 R alpha	IL-6 R	IL-8
6	ICAM-1	ICAM-3	IGFBP-3	IGFBP-6	IGF-I SR	IL-1 R4/ST2	IL-1 RI	IL-11	IL-12p40	IL-12p70	IL-17	IL-2 R alpha	IL-6 R	IL-8
7	I-TAC	Lymphotactin	MIF	MIP-1 α	MIP-1 β	MIP-3 β	MSP-α	NT-4	Osteoprotegerin	Oncostatin M	PIGF	sgp130	sTNF RII	sTNF-RI
8	I-TAC	Lymphotactin	MIF	MIP-1 α	MIP-1 β	MIP-3 β	MSP-α	NT-4	Osteoprotegerin	Oncostatin M	PIGF	sgp130	sTNF RII	sTNF-RI
9	TECK	TIMP-1	TIMP-2	Thrombopoletin	TRAIL R3	TRAIL R4	uPAR	VEGF	VEGF-D	Blank	Blank	Blank	Blank	POS
10	TECK	TIMP-1	TIMP-2	Thrombopoletin	TRAIL R3	TRAIL R4	uPAR	VEGF	VEGF-D	Blank	Blank	Blank	Blank	POS

RayBio Human Cytokine Antibody Array 8 (54)

	a	b	c	d	e	f	g	h	i	j	k	l	m	n
1	POS	POS	NEG	NEG	BLANK	Activin A	ALCAM	B7-1(CD80)	BMP-5	BMP-7	Cardiotrophin-1	CD14	CXCL-16	DR6(TNFRSF21)
2	POS	POS	NEG	NEG	BLANK	Activin A	ALCAM	B7-1(CD80)	BMP-5	BMP-7	Cardiotrophin-1	CD14	CXCL-16	DR6(TNFRSF21)
3	Endoglin	ErbB3	E-Selectin	Fas Ligand	ICAM-2	IGF-II	IL-1 R II	IL-10 R beta	IL-13 R alpha 2	IL-18 BP alpha	IL-18 R beta	MMP-3	IL-2 R beta	IL-2 R gamma
4	Endoglin	ErbB3	E-Selectin	Fas Ligand	ICAM-2	IGF-II	IL-1 R II	IL-10 R beta	IL-13 R alpha 2	IL-18 BP alpha	IL-18 R beta	MMP-3	IL-2 R beta	IL-2 R gamma
5	IL-21R	IL-5 R alpha	IL-9	IP-10	LAP	Leptin R	LIF	L-Selectin	M-CSF R	MMP-1	MMP-13	MMP-9	MPIF-1	NGF R
6	IL-21R	IL-5 R alpha	IL-9	IP-10	LAP	Leptin R	LIF	L-Selectin	M-CSF R	MMP-1	MMP-13	MMP-9	MPIF-1	NGF R
7	PDGF AA	PDGF-AB	PDGF R alpha	PDGF R beta	PECAM-1	Prolactin	SCF R	SDF-1beta	Siglec-5	TGF-alpha	TGF beta .2	Tie-1	Tie-2	TIMP-4
8	PDGF AA	PDGF-AB	PDGF R alpha	PDGF R beta	PECAM-1	Prolactin	SCF R	SDF-1beta	Siglec-5	TGF-alpha	TGF beta .2	Tie-1	Tie-2	TIMP-4
9	VE-Cadherin	VEGF R2	VEGF R3	BLANK	BLANK	BLANK	BLANK	BLANK	BLANK	BLANK	BLANK	BLANK	BLANK	POS
10	VE-Cadherin	VEGF R2	VEGF R3	BLANK	BLANK	BLANK	BLANK	BLANK	BLANK	BLANK	BLANK	BLANK	BLANK	POS
