# Supplementary material for: Stromal Cells Positively and Negatively Modulate the Growth of Cancer Cells: Stimulation via the PGE2-TNFα-IL-6 Pathway and Inhibition via Secreted GAPDH-E-Cadherin Interaction
Source: PLoS One. 2015 Mar 18;10(3):e0119415. doi: 10.1371/journal.pone.0119415 (PMC4364666; doi:10.1371/journal.pone.0119415)
Supplement: S19 Fig — (A) Effect of recombinant GAPDH on MKN-7 cells. MKN-7 cells were cultured with human recombinant wtGAPDH at 15 μg/ml for 1 day. The cells were fixed under cell-permeabilized conditions, immunostained with anti-FLAG and anti-mouse IgG1 Alexa Fluor 546 antibodies, and analyzed by confocal microscopy. Scale bar is 20 μm. (B) MKN-7 cells were cultured with human erythrocyte GAPDH at 5 U/ml for 1 day (left). The cells were fixed under nonpermeabilizing conditions and stained with the indicated antibodies. Scale bars are 50 μm. (PDF) [file pone.0119415.s019.pdf]

Figure S19

A

Lateral side

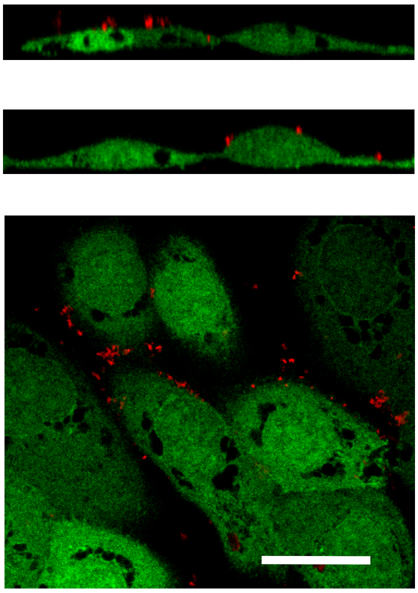

Green:GFP
Red:anti-FLAG

B

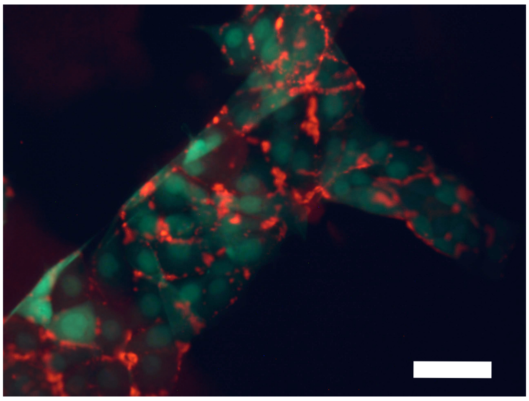

Green:GFP
Red:anti-GAPDH

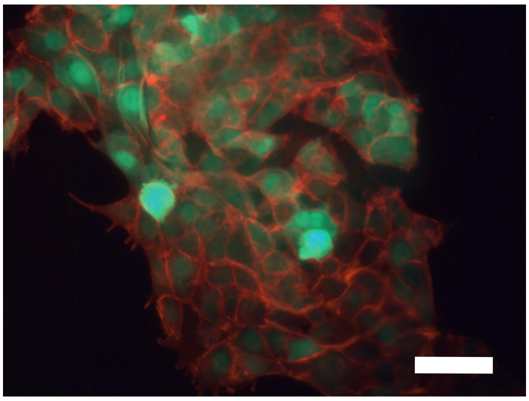

Green:GFP
Red:anti-E-cadherin
