# Supplementary material for: Stromal Cells Positively and Negatively Modulate the Growth of Cancer Cells: Stimulation via the PGE2-TNFα-IL-6 Pathway and Inhibition via Secreted GAPDH-E-Cadherin Interaction
Source: PLoS One. 2015 Mar 18;10(3):e0119415. doi: 10.1371/journal.pone.0119415 (PMC4364666; doi:10.1371/journal.pone.0119415)
Supplement: S22 Fig — Tissue sections of human gastric cancer and GIST were stained with anti-phospho-STAT3. Numbers in parentheses are percent. Representative photos of the immunostained sections are shown. (PDF) [file pone.0119415.s022.pdf]

Figure S22

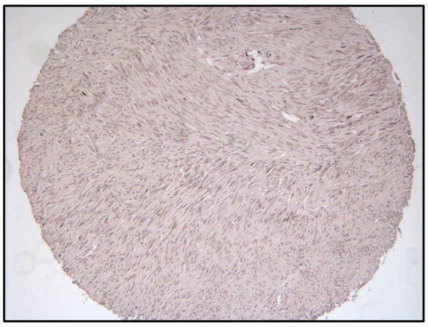

57F GIST

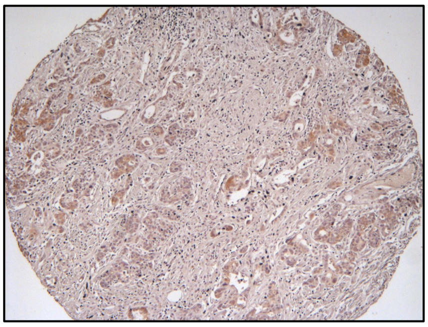

66M adenocarcinoma IIIB

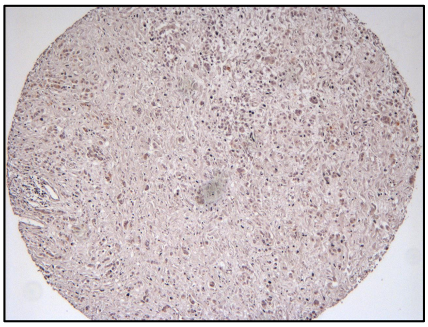

52F signet ring cell carcinoma IIIB

		p-STAT3 (%)		
	Total	Negative	Weakly positive	Positive
GIST	7	7 (100.0)	0 (0.0)	0 (0.0)
Adenocarcinoma	19	6 (31.6)	7 (36.8)	6 (31.6)
Signet ring cell carcinoma	14	7 (50.0)	5 (35.7)	2 (14.3)
